# Torus-margo pits cannot function in vessel-bearing angiosperms

**DOI:** 10.1038/s42003-026-09800-x

**Published:** 2026-04-06

**Authors:** Maciej A. Zwieniecki, Aude Tixier, Jessica Orozco, Keunhwan Park, Anneline H. Christensen, Sif Fink Arnbjerg-Nielsen, Kaare H. Jensen

**Affiliations:** 1 Plant Sciences, UC Davis, Davis, CA USA; 2 Univversity Clermont Auvergne, INRAE, PIAF, Clermont-Ferrand, France; 3 Stanford Doerr School of Sustainability, Stanford University, Stanford, CA USA; 4 Department of Mechanical Engineering, Gachon University, Seongnam, South Korea; 5 Department of Physics, Technical University of Denmark, Lyngby, Denmark

**Keywords:** Plant evolution, Abiotic, Plant physiology

## Abstract

The evolution of plant hydraulics reflects a trade-off between efficiency and safety, between maximizing water transport while preventing embolism spread. Two major lineages achieved this balance differently: conifers and Gingko optimized safety through low resistance torus-margo pits, while angiosperms maximized hydraulic efficiency through long, wide vessels. It is therefore striking that no lineage evolved a “hybrid” design combining the efficiency of vessels with the low resistance of margo membranes with potential exception of Ephedraceae. Here we use a combination of experimental and theoretical approaches to show that the geometry of vessels inherently limits the pressure differentials required for torus-margo function.

## Abstract (web summary)

Mechanistic modelling identifies conduit geometric constraints on torus-margo membrane function, resolving the surprising absence of this low-resistance, high-safety design in vessel-bearing plants.

## Introduction

Efficient water transport is fundamental to plant survival, supporting photosynthesis, growth, and resilience across diverse environments. Despite this shared imperative, the vascular architectures of conifers and angiosperms have diverged sharply. Based on conduit anatomy alone, conifers would seem to be at a disadvantage: their tracheids are short, narrow, and lack perforation plates, leading to higher hydraulic resistance. Angiosperms, in contrast, evolved wide vessels with perforation plates, reducing both lumen and end-wall resistance, an arrangement widely regarded as optimized for rapid, efficient water flow^1^.

Surprisingly, conifer tracheids and angiosperm vessels exhibit comparable hydraulic conductance, both at the individual conduit level and across the sapwood area, despite stark anatomical differences^2^. This convergence raises an interesting question: how do conifers remain hydraulically competitive despite less favorable conduit geometry? One key lies in their bordered pits, specifically, the torus-margo design unique to conifers and a few other gymnosperm lineages. A torus-margo pit features a thickened impermeable central region (torus) suspended by a spoke-like network of flexible fibrils forming a highly porous margo. Under normal conditions, the margo’s large pores allow for relatively unhindered water flow; should an embolism form, the torus seals against the pit aperture, isolating the gas-filled conduit and preventing embolism spread into neighboring conduits^3^. This architecture confers exceptionally low hydraulic resistance while providing an effective mechanism for embolism isolation. In contrast, angiosperms typically rely on homogenous pit membranes, densely woven networks of microfibrils that impede embolism propagation, but at the cost of substantially greater resistance.

Considering that the torus-margo design reduces pit-area resistance by nearly 59-fold compared to angiosperm pit membranes while maintaining embolism safety, it is puzzling that this architecture is absent from vessel-to-vessel transport and entirely missing in the highly dominant angiosperms^2^. Why don’t angiosperms have torus-margo pit membranes? In theory, combining the low lumen resistance of vessels with the low pit-membrane resistance of torus-margo structures could yield the best of both worlds**:** exceptionally high-water transport efficiency coupled with robust embolism protection. Interestingly, the only reports of such an arrangement come from early anatomical descriptions of *Ephedra*; however, their presence remains uncertain, as later studies emphasize torus-margo pit occurrence in tracheids rather than vessels^4–8^.

Here, we investigate the surprising absence of torus-margo membranes in vessel-bearing plants. Understanding this phenomenon requires quantifying the forces necessary to displace the torus into its closed, or aspirated, position. To this end, we experimentally estimate the pressure gradient required to induce pit aspiration. We then evaluate whether the generation of dynamic pressures sufficient to deflect the torus is constrained by vessel geometry, and whether such limitations could explain why the torus-margo design is absent in vessels.

**Fig. 1:**
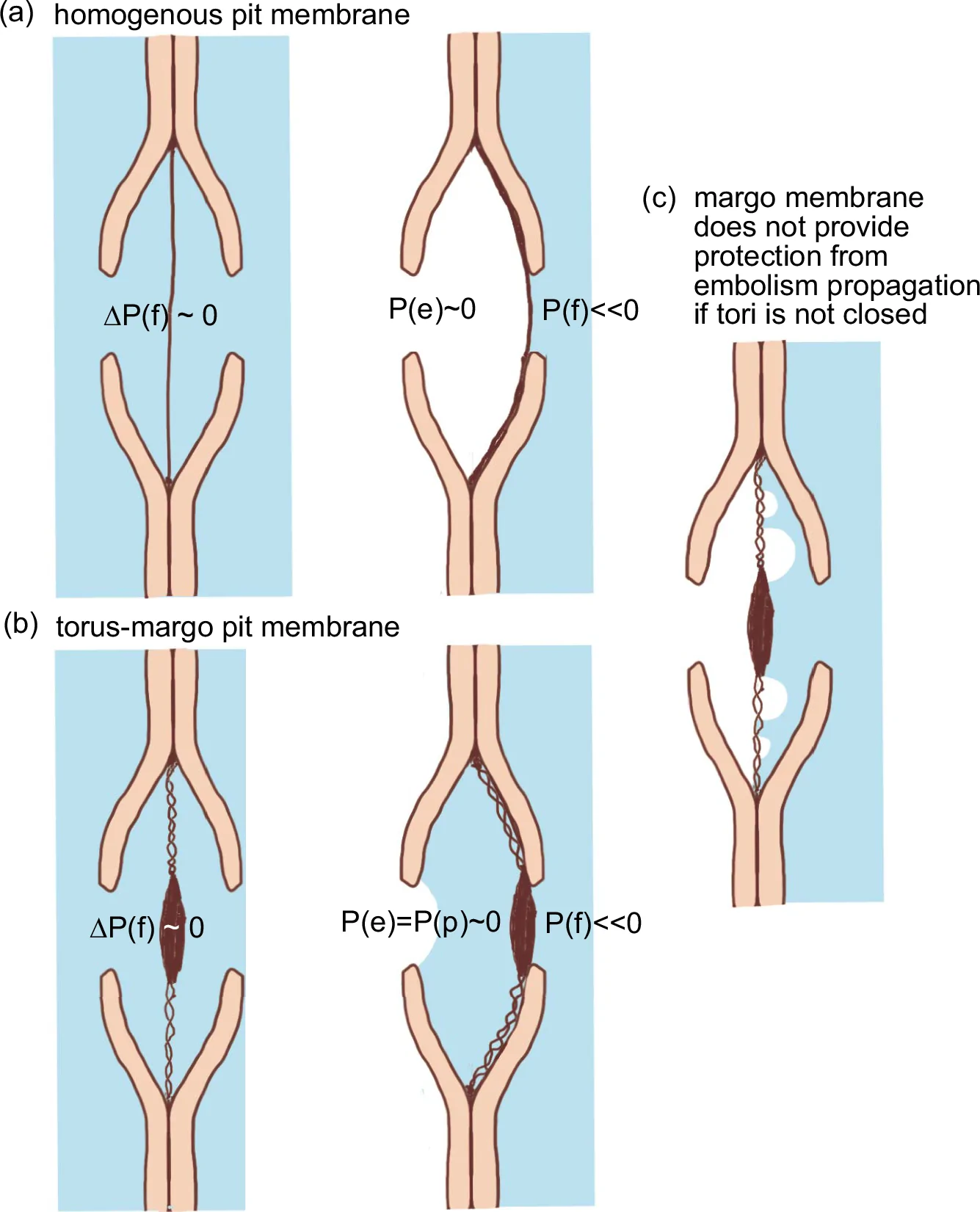
Conceptual schematic illustrating pit membrane function. **a** In the functional (water-filled) state, the pressure gradient across the homogeneous pit membrane is minimal (ΔP(f) ~ 0), allowing water to move freely through the membrane. Following embolization, an air water interface forms on the microfibril mesh, and the resulting pressure differential (P(e)»P(f) can deform the membrane. **b** In a torus-margo pit, a small pressure gradient exists under functional conditions. During embolism, the torus must be deflected and pressed against the pit aperture before the advancing air-water interface reaches the margo, thereby sealing the pit and preventing air entry into adjacent conduits. **c** If, during embolism, the pressure differential is insufficient to displace the torus into its sealed position, the air-water menisci within the margo pores fail to sustain tension, permitting air seeding between conduits, thereby allowing the embolism to propagate.

A key premise of our approach is that pit aspiration must occur *before* the air-water interface reaches the margo, as only preemptive torus sealing can prevent air seeding into adjacent conduits given the high porosity of the margo (conceptually illustrated in Fig. 1). This implies that torus displacement is not driven directly by capillary suction at the margo pores, but rather by transient pressure differences developing between embolized and water-filled conduits. We therefore consider a scenario in which these pressure differences arise from the hydrostatic forces generated as an embolus expands and displaces water within the conduit network. However, this displacement of water introduces and additional constraint. The fluid forced out of an embolizing conduit must enter neighboring conduits, where it can transiently increase local pressure. This effect would reduce the very pressure gradient that drives torus closure, effectively damping the differential required for aspiration. In summary, we test two hypotheses^1^: that embolus expansion can generate pressure differences capable displacing the torus into its sealed position, and^2^ that vessel geometry suppresses this mechanism by reducing the pressure gradient available for torus aspiration.

## Results and discussion

### Quantification the pressure differential required for torus closure

**Fig. 2:**
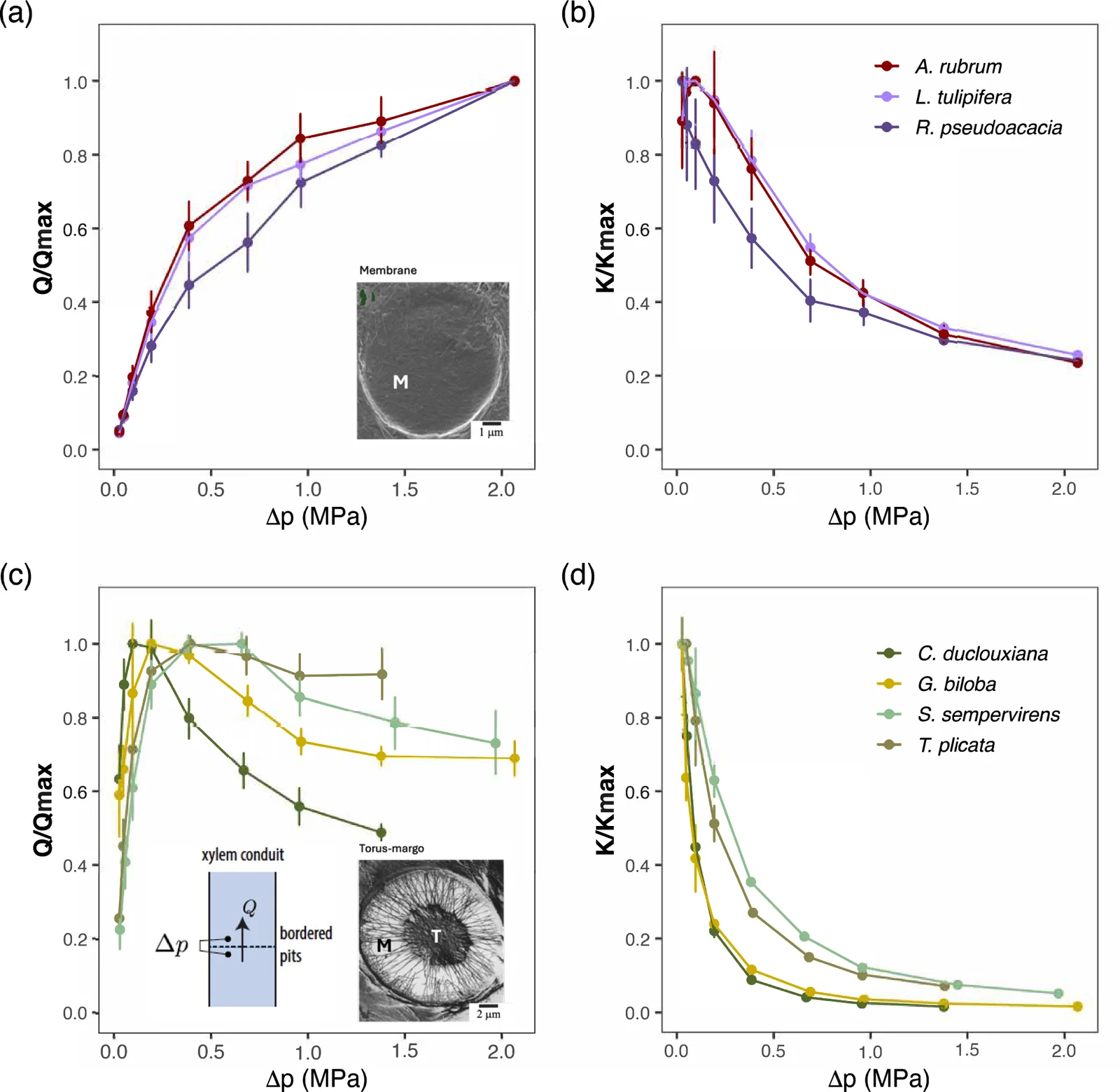
Pressure-dependent flow and conductance across bordered pits. **a**–**d** Relationship between applied pressure difference (Δp), flow rate (*Q*), and hydraulic conductance (K=Q/Δp) for angiosperms with homogeneous pit membranes (**a**, **b**) and gymnosperms bearing torus-margo pits (**c**, **d**). Panels (**a**) and (**c**) show flow rate as a function of applied pressure, while (**b**) and (**d**) show the corresponding pressure-conductance relationships. Data represent flow across a single layer of bordered pits from three angiosperm and four gymnosperm species, with *Q* and K normalized by their respective maximum values. (5 samples per species, error bars indicate SD). In torus-margo membranes (**c**), pressures in the range of $\Delta {p}_{\min }=0.1-0.5$ MPa induce progressive closure, resulting in a strong decline in flow. We note that elements of the data shown in (**c**) appear in previous reports by our team, highlighting the insensitivity of the flow variations in the input pressure as an example of a programmable flow controller^29^. Insets chow meaning of Δp (**c**), and micrographs of homogeneous membrane (**a**) and torus margo (**c**)^30^.

We first examined the hydraulic behavior under controlled pressure gradients. Specifically, we measured the relationship between pressure drop (Δp) and fluid flow rate (*Q*) across xylem conduit-conduit interfaces, allowing us to estimate the actuation pressure necessary for pit aspiration. This experiment provides a reference point for assessing whether the pressure gradients generated during embolus expansion could realistically achieve closure. Data from 7 species (4 gymnosperm species^8,9^ and 3 angiosperms) were recorded (Fig. 2). At low pressures, the flow rate (Q/Q_max_) increased across all species, consistent with passive fluid movement through open pits. However, even within this low-pressure range, the relative conductance (K/K_max_) declined continuously. This behavior is inconsistent with the expectation for a system of parallel cylindrical conduits connected by non-deflected membranes, where low-Reynolds-number theory predicts a linear relationship between flow and pressure according to the Hagen-Poiseuille (and Darcy) law^10^. The observed loss of conductance suggests that bordered pits begin to respond to increasing pressure differentials even at minimal pressure drops (~0.1 MPa) in both angiosperms and conifers.

At higher pressures, a striking divergence between conifers and angiosperms species emerged. In angiosperms, flow continued to increase monotonically with pressure, showing a slightly sub-linear trend that may reflect bending of the homogenous membrane against the pit aperture or bulk tissue deformation^11,12^. In contrast, torus-bearing species exhibited a different behavior: with distinct maximum pressure beyond which the flow rate decreases. This phenomenon occurred at pressures in the range Δp=0.1−0.5 MPa. We attribute this nonlinearity to progressive torus displacement and sealing of pit apertures. For subsequent analyses, we defined the actuation pressure, the pressure differential required to induce 80% loss of conductance as $(\Delta {p}_{\min } \approx 0.25\,{\mbox{ to }}\,0.75\,{{{\rm{MPa}}}})$. This range is consistent with our previous first-principles estimate ~0.15 MPa^13^ and aligns with values reported for pit-sealing pressures in conifers, which typically span ~ 0.05–1 MPa^14–17^.

When, if ever, do such threshold inter-conduit pressure differentials $(\Delta {p}_{\min } \approx 0.25\,{\mbox{ to }}\,0.75\,{{{\rm{MPa}}}})$ arise within the xylem? Even under hydraulic duress, near P_50_, the water potential at which 50% of conductivity is lost due to embolism (≈−2.9 MPa in angiosperms; ≈−5.0 MPa in conifers^18^;), the pressure difference between functional conduits remains far below this threshold. The overall pressure drop driving water transport is effectively distributed along the entire axial series of conduits, such that the pressure drop across any single pit interface approximates the bulk xylem pressure $({p}_{x})$ divided by the number of conduits (N) along the pathway. In a 50 m conifer with 1 mm long tracheids ($N \approx {10}^{4}$), this yields an inter-conduit pressure difference of about ${10}^{-4}\,$MPa, while in an angiosperm tree of similar height (conduit length ≈ 10 cm, $N \approx {10}^{2}$) it is roughly ${10}^{-2}$ MPa, two to three orders of magnitude below the $\Delta {p}_{\min }$ required for torus aspiration. Thus, even during significant loss of hydraulic function, normal pressure distributions in the xylem cannot generate the differentials needed for closure; such pressure differences, however, can develop during embolism propagation.

### Assessment of transient pressure differences generated during embolism propagation

To assess whether transient pressure differences generated during embolism propagation can produce forces sufficient to close the torus-margo, we model the velocity of the advancing gas-liquid interface in a water-filled conduit (tracheid or vessel). As the gas phase expands, the liquid is displaced through the bordered pits into adjacent conduits; the driving pressure is the net drop across the embolizing segment. As the gas bubble grows, it pushes water ahead of the advancing gas-liquid interface, forcing flow through the pit membranes. The resulting motion can be described by adapting the classical Washburn (1921) model for viscous flow in a capillary under an imposed pressure difference. In this formulation, the velocity of the advancing interface (dl/dt) reflects the balance between the driving pressure across the meniscus (ΔP) and the viscous resistance of the liquid column:

1 $$\frac{{dl}}{{dt}}=\frac{\Delta {{{\rm{P}}}}}{8{r}^{2}\eta l}({r}^{4}+4\epsilon {r}^{3})$$

Where *r* the conduit radius (m), *η* the viscosity of water (Pa·s), l the conduit length (m), and ϵ the slip length. Under no-slip conditions (ϵ « *r*), the slip term is negligible, and the expression reduces to:

2 $${v}_{e}=\frac{{r}^{2}\Delta {{{\rm{P}}}}}{8\eta l}$$

Which gives the velocity of the advancing meniscus (m s^−1^). From this, the velocity of water passing through the pit membrane (*v*_*pit*_) can be expressed as:

3 $${v}_{{pit}}={v}_{e}{f}_{{Ap}/{Ac}}\frac{{A}_{{cw}}}{{A}_{{ccs}}}$$

Where ${f}_{{Ap}/{Ac}}$ is the fraction of the conduit wall occupied by bordered pits (0.0728 for conifers^19^ and 0.0616 for angiosperms^20^), ${A}_{{cw}}$ is the conduit wall area ($2{\pi }{rl}$), and ${A}_{{ccs}}$ is the conduit cross-sectional area (${\pi }r$^2^).

The dynamic pressure acting on the torus as displaced water flows through the pit membrane is then estimated as:

4 $${P}_{d}=\frac{1}{2}\rho {{v}_{{pit}}}^{2}$$

Where ρ is the density of water (kg/m^3^). The dynamic pressure term (Eq. (4)) represents the local, reversible conversion of static pressure into kinetic energy as flow accelerates through the pit aperture, consistent with Bernoulli’s principle. This pressure does not constitute an additional or irreversible pressure loss across the pit. Static pressure is recovered as flow decelerates downstream of the aperture. Accordingly, the model assumes that pit chamber pressure is governed by the imposed pressure difference between adjacent conduits, while the dynamic pressure term captures the transient force exerted on the torus during flow acceleration. The total force exerted on the torus, approximated as a circular plate of radius *R*_T_ = 3 μm^21^, is:

5 $${F}_{t}={P}_{d}{A}_{T}$$

where *A*_T_ = πR_T_^2^.

**Fig. 3:**
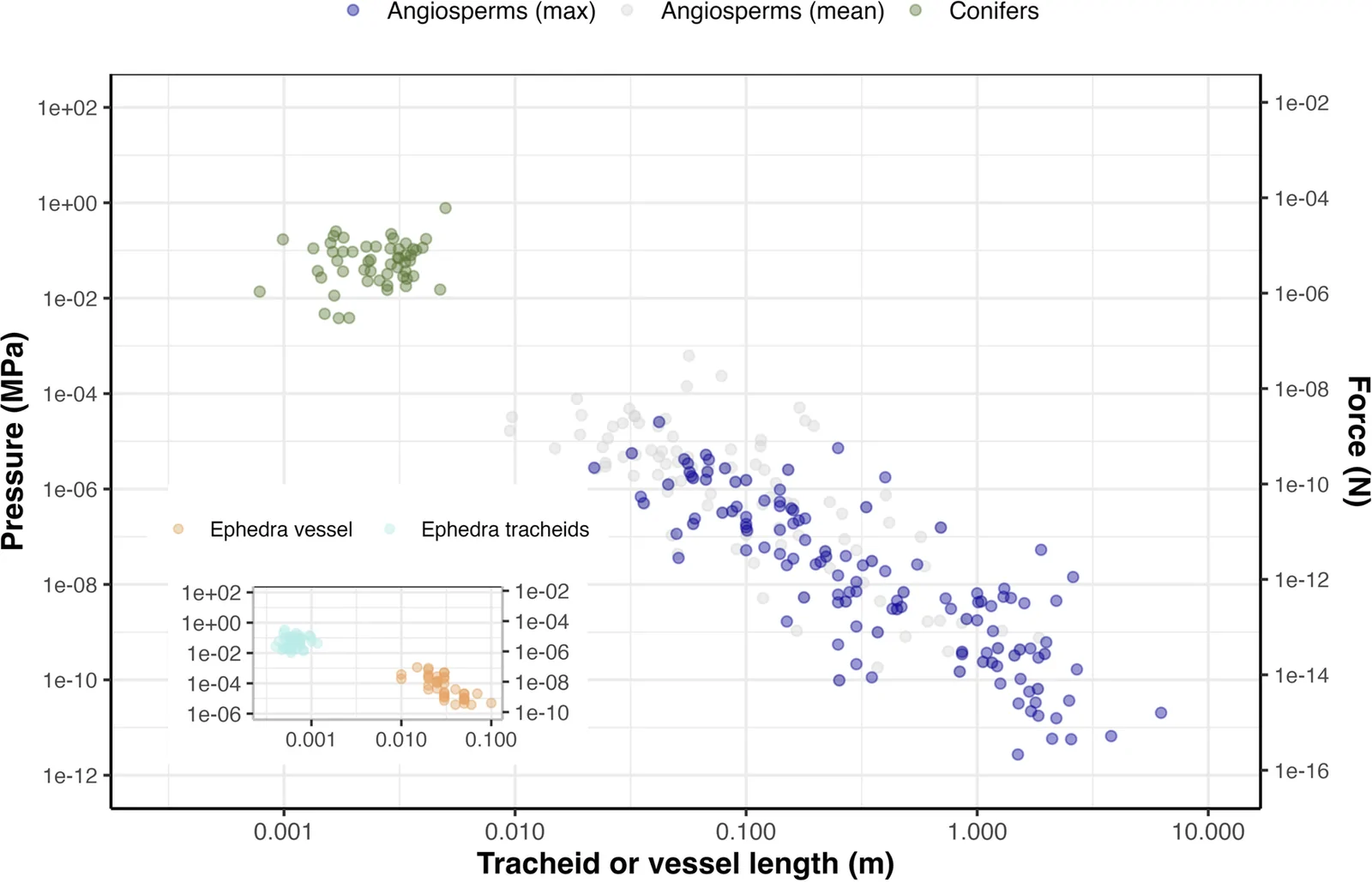
Dynamic pressure and force on torus–margo pits as a function of conduit length. Estimated dynamic pressure (MPa) and corresponding force (N) acting on the torus as a function of tracheid or vessel length (m). Dynamic pressures were calculated using Eq.,(4) assuming bordered pits cover 7.28% of conduit wall area in conifers’ stems^19^ and 6.16% in angiosperms^20^. Forces were estimated for a torus with a radius of 3 μm^21^. Green symbols represent conifers (*n* = 60), purple and gray represent angiosperms using maximum and mean vessel length values (*n* = 181), respectively. Inset: comparison of *Ephedra* tracheids (blue) and vessels (orange) (*n* = 36). Data in Supplemental Material Table 1.

Our estimates (Fig. 3) show that embolus expansion in conifer tracheids can generate on average dynamic pressures of 0.0903 MPa (ranging from 0.004 to 0.77 MPa), corresponding to average force of 2.55 μN (ranging from 0.1 to 21.8 μN), values consistent with experimental measurements of torus deflection^22^. In contrast, angiosperm vessels produce pressures only in the range of 2.72 μPa–25.5 Pa (forces: 0.077 fN −17.6 nN), several orders of magnitude below the threshold for torus closure^22^. Thus, findings indicate that only tracheid-scale conduits can generate sufficient pressure differentials for effective torus aspiration. *Ephedra* occupies an intermediate position. Its tracheids produce average pressure 0.075 MPa (in the range of 0.03–0.34 MPa); average force 2.13 μN (in the range of 029–9.6 μN) comparable to conifers, consistent with aspiration, whereas its vessels generate average pressure of 0.19 kPa (ranging from 3.88 Pa to 1.14 kPa; average force of 5.49 nN (in range of 0.11 nN to 32.3 nN) far too low for torus aspiration. This suggests that torus-margo pit membranes, if present in *Ephedra* vessels, would be mechanically nonfunctional.

### Pressure modulation caused by the expelled-volume effect during bubble expansion

Apart from the dynamic pressures generated during embolus expansion, we must also consider an accompanying process that may influence the function of the torus-margo pit membrane. As the embolus expands, the displaced volume is forced into adjacent conduits, raising their internal pressure in proportion to the added volume^23^. This increase partially offsets the pressure difference between the embolizing conduit and its water-filled neighbor, thereby reducing the net driving gradient. Consequently, the inter-conduit pressure differential may drop below the threshold required for torus closure before the advancing air-water interface reaches the pit membrane.

**Fig. 4:**
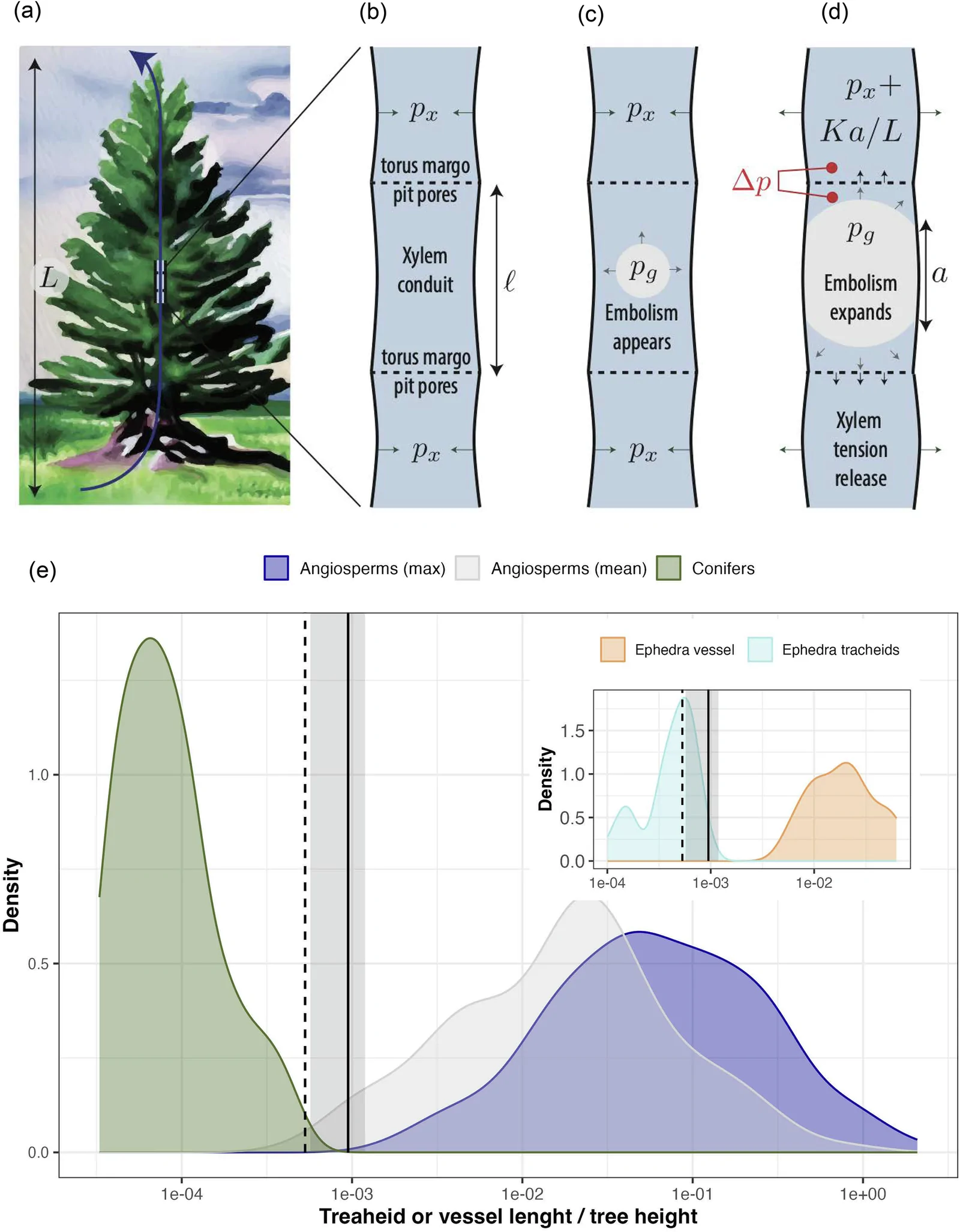
Conceptual model of embolism expansion and inter-conduit pressure constraints on torus–margo pit function. **a** Sketch of a conifer tree depicting the total hydraulic pathway length *L*, representing the average distance traveled by a water molecule moving in the xylem from root to shoot (blue arrow). **b** Close-up view of a vertical series of xylem conduits under normal flow conditions. The xylem pressure (*p*_*x*_) varies minimally between adjacent conduits, with absolute values depending on species and conditions (Supplemental Material Table 2). Because xylem pressure is below atmospheric pressure, the tissue is pulled slightly inwards. **c**, **d** Sequence illustrating embolism initiation and expansion. When a small embolism forms, the gas-phase pressure (${p}_{g}\approx 0$ Pa) exceeds that of the surrounding liquid. The pressure difference between the bulk xylem sap and the embolism causes the gas pocket to expand, thus increasing its length (a) over time. The displaced liquid flows axially along the xylem, releasing tension in the upstream and downstream conduits by an amount $K\ell {{/}}L$, where K is the bulk modulus. This process reduces the inter-conduit pressure difference $\Delta {{{\rm{p}}}}$ (see Eq. (6)). Torus-margo pit closure (aspiration) can only occur if $\Delta {{{\rm{p}}}}$ exceeds the actuation threshold $\Delta {p}_{\min }\approx 0.25{to}0.75\,$MPa, which defines a maximum conduit length ${\ell }_{\max }$ (Eq. (7)). **e** Distribution of relative conduit length $({\ell }_{\max }/L)$ in conifers and angiosperms showing how conduit geometry aligns with the model-predicted threshold for torus aspiration. The vertical axis describes the probability density function (PDF) based on anatomical measurements from 60 conifer species and 157 angiosperm species (maximum vessel length; mean length from 159 species; Supplemental Material Table 1). Green shading indicates conifers, purple shading angiosperms (maximum vessel length), and gray shading angiosperms (mean vessel length). The gray vertical band denotes model-predicted transition region where torus aspiration becomes unstable (Supplemental Material Table 3). The solid vertical line marks the model-predicted critical threshold for conifers, ${\ell }_{\max }/L\approx 9.4\times {10}^{-4}$, while the dashed vertical line represents the corresponding threshold for angiosperms ${\ell }_{\max }/L\approx 5.3\times {10}^{-4}$. Inset: Comparison of *Ephedra* tracheids and vessels (*n* = 36). Density distributions show that tracheids (blue) fall within the functional range predicted by the model (below the shaded threshold region), while vessels (orange) exceed this range. (Supplemental Material Table 1).

To quantify this pressure modulation caused by the expelled-volume effect, we developed a simple model of bubble expansion to examine the dynamics of pressure equilibration and identify the conditions that allow torus-margo pit membranes to function within whole-plant hydraulic constraints. The scenario (Fig. 4 a–d) begins with the xylem fluid at equilibrium under tension, at characteristic pressures for angiosperms and conifers at P_50_. When cavitation occurs, whether by meniscus failure, expansion of a nanobubble, or embolism formation via heterogeneous nucleation, a gas pocket forms^24^. Initially composed primarily of water vapor, the gas phase has a positive pressure (*p*_*g*_ ≥ 0 MPa); the pressure difference between the liquid and the gas phase causes the bubble to expand. As the bubble grows, liquid is expelled through the bordered pits into adjacent conduits, increasing their xylem pressure $({p}_{x})$ by an amount proportional to the bubble length ℓ and the elastic properties of the tissue. The resulting pressure can be expressed as ${p}_{x}+K\ell {{/}}L$, where $K\approx 5\,{{{\rm{Gpa}}}}$ is the bulk modulus^25^ of the sap and xylem tissue, and *L* is the total flow path length (i.e., the plant height). Water movement is assumed to occur primarily along axial pathway, consistent with high radial resistance reported for vessel-bearing species^26,27^.

During embolus expansion, the pressure difference Δp across the conduit-conduit interface:

6 $$\Delta p={p}_{g}-({p}_{x}+K\ell /L)$$

decreases as the gas pocket length ℓ increases, because the neighboring conduit’s pressure increases. Based on our experimental results, a minimum pressure difference $\Delta {p}_{\min }=0.25\,{{\mathrm{to}}}\,0.75\,{{\mathrm{Mpa}}}$ is required to reduce conductance by 80%, corresponding to closure of most torus-margo pits. Critically, the closing process must occur before the gas bubble reaches the pit membrane, that is, before $\ell {{=}}{\ell }_{\max }$, where ${\ell }_{\max }$ is the conduit length.

Assuming these limiting conditions ($\Delta p=\Delta {p}_{\min }$ and ($\ell {{=}}{\ell }_{\max }$) in Eq.(6) yields the maximum relative conduit length:

7 $$\frac{{\ell }_{\max }}{L}=\frac{{p}_{g}{-\Delta {p}_{\min }-p}_{x}}{K}$$

This relationship shows that conduits whose relative length $({\ell }_{\max }/L)$ exceeds ~ 5.3 × 10^−4^ in angiosperms or ~ 9.47 × 10^−4^ in conifers cannot close their torus-margo pits before the advancing meniscus reaches the membrane. In other words, xylem conduits cannot be simultaneously long and exploit the torus-margo mechanism for effective embolism isolation.

To test this prediction, we compared conduit length relative to tree height across a broad dataset of angiosperm and gymnosperm species (Fig. 4e). The resulting distributions show a clear separation between the two groups near the model-predicted threshold for torus aspiration. Conifers exhibit relative conduit length centered around ${\ell }_{\max }/L\approx 4\times {10}^{-5}$, well below the critical threshold, whereas angiosperms peak near ${\ell }_{\max }/L\approx 4\times {10}^{-2}$, far above it (model parameters in Supplemental Material Tables 2 and 3). When maximum vessel lengths are used, the separation between angiosperms and conifers is complete. Using maximum length values is warranted as the inability to compartmentalize embolisms within a few long vessels could result in the spread of embolism to adjacent shorter conduits. Using mean vessel lengths introduces a slight overlap between groups, suggesting that aspiration could, in principle, persist in some species with unusually short vessels. However, this would require developmental coordination between pit membrane architecture and vessel length. As shown in the inset of Fig. 4e, Ephedra tracheids occupy the conifer-like region of the distribution, whereas its vessels cluster among angiosperms. This contrast mirrors the modeled mechanical limits: tracheid-scale conduits fall within the feasible range for aspiration, while vessel-scale conduits exceed it.

The torus-margo pit membrane represents a defining innovation in conifer hydraulics, combining low hydraulic resistance with effective embolism isolation. Given these functional advantages, the absence of torus-margo pit membranes in angiosperm conduits presents a surprising evolutionary conundrum. The paradox deepens when considering that several drought-resistant angiosperms with relatively short vessels have independently evolved torus-like pit structures, yet these lack the highly porous margo^28^, suggesting that the torus-margo design remains within evolutionary reach. *Ephedra* presents a particularly intriguing case in this context. It is the only known genus reported to possess torus-margo pit membranes in its vessels, based on historical anatomical descriptions. However, subsequent analyses have largely restricted descriptions of torus-margo structures to tracheids, providing no definitive evidence of their occurrence or operation in *Ephedra* vessels. Our results help elucidate on this ambiguity, suggesting that torus-margo pit membranes are unlikely to occur, or, if present, are nonfunctional in *Ephedra* vessels.

## Conclusions

Here, we propose two non-exclusive explanations for why torus-margo pit membranes never evolved, or failed to persist, in vessel-bearing angiosperms. The first centers on the dynamics of force generation required to aspirate the torus before the advancing air-water interface reaches the porous margo (Fig. 3). In conifers, short tracheids enable embolus expansion to generate sufficient dynamic pressure to fully aspirate the torus. In contrast, the slower fluid velocities in long vessels produce forces far too weak to achieve aspiration, rendering the mechanism ineffective for embolism isolation. Second, the larger volume of water displaced during embolism expansion in vessels introduces a back-pressure effect, further reducing the dynamic pressure across the pit membrane and limiting the force available for torus displacement.

Even among conifers, where the mechanism evolved and persists, its functionality depends on a few morphological traits to consider.

1. Dynamic pressure is highly dependent on the ratio of pit membrane area to total conduit wall area $({f}_{{Ap}/{Ac}})$; lower the fractions generate higher pressure. This raises the question on maximum functional ${f}_{{Ap}/{Ac}}$ and, by extension, the number of bordered pits that still allow the torus to aspirate before the advancing air-water meniscus reaches the membrane. It’s therefore not surprising that conifers have ~10 times lower ${f}_{{Ap}/{Ac}}$ than angiosperms, as this low fraction is necessary for effective embolism compartmentalization.
2. Similarly, the force generated on the torus depends strongly on its radius, with smaller tori experiences lower forces. This raises questions about torus size relative to dynamic pressure. It is possible that torus size is inversely correlated with dynamic pressure, i.e., shorter and wider tracheids may possess smaller tori.
3. Increasing wall stiffness and level of hydraulic connectives among conduits can produce a backlash effect, reducing the velocity of the expanding embolus and significantly lowering the dynamic pressure, particularly in small/young conifers with low $\ell {{/}}L$ values.

The torus-margo membrane represents one of the most effective nature solutions to the efficiency-safety trade-off in plant hydraulics. Yet, our findings reveal that its sealing function is constrained by the very geometry that enables vessel-based efficiency.

## Methods

### Plant material

Twigs (~6–7 mm in xylem diameter) of Sequoia sempervirens (D. Don) Endl., Cupressus duclouxiana Hickel, Ginkgo biloba L., Thuja plicata Donn ex D. Don, Liriodendron tulipifera L., Robinia pseudoacacia L., and Acer rubrum L. were sampled in the vicinity of the UC Davis Arboretum and brought directly to the laboratory for anatomical and hydraulic analyses. Conduit length distribution for S. sempervirens, C. duclouxiana, G. biloba, and T. plicata was estimated on longitudinal radial sections using light microscopy. Images were analyzed with Fiji (ImageJ). Average tracheid length was estimated from ~20 to 30 measurements. Estimates of tracheid length were used to determine sample length to avoid premature opening of conduits.

### Pressure–flow measurements

**Fig. 5:**
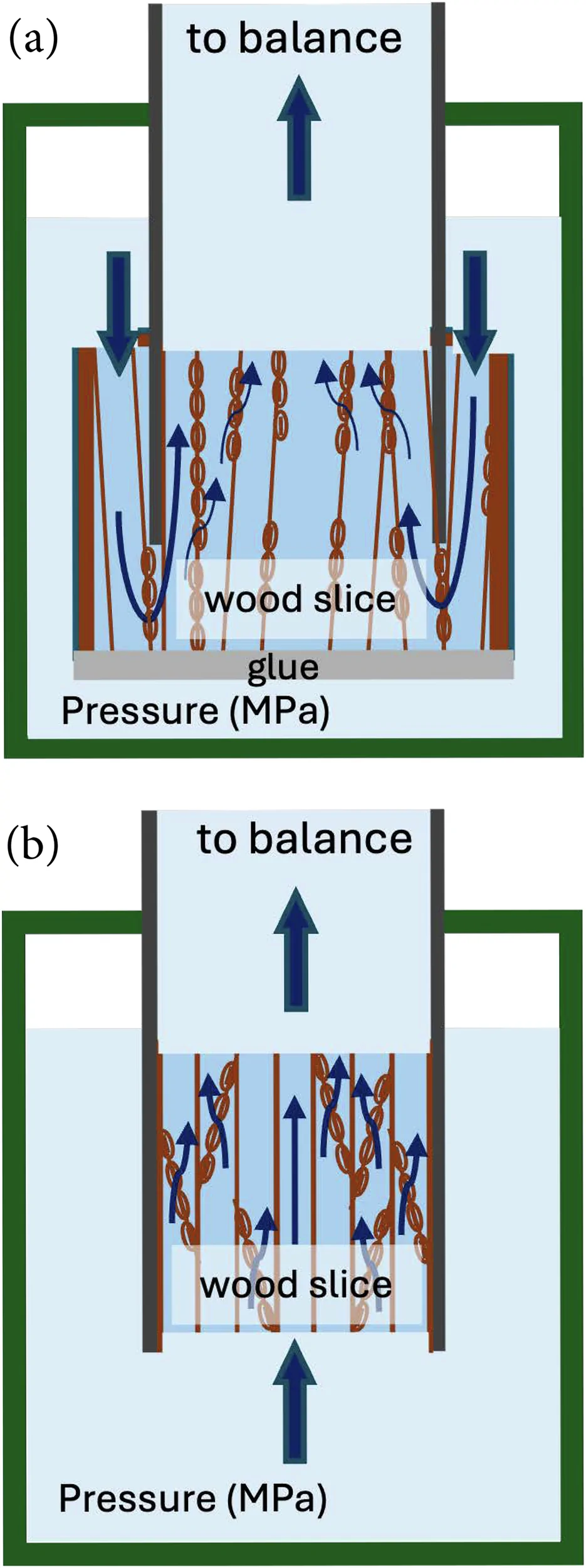
Schematic of experimental measurement. **a** schematic of the experimental setup used to measure flow across a single layer of angiosperm pit membranes under variable pressure, **b** schematic of the corresponding setup for torus-margo membranes. Blue arrows depict water flow path in both setups.

Determination of the pressure–flow relationship for vessel-bearing species was conducted on five twigs per species using 2 cm long samples. The basal end of each sample was hydraulically blocked using superglue that covered the entire cross section. The proximal end was then forced onto a metal tube (5 mm diameter) to a depth of approximately 3–4 mm, such that part of the xylem (0.5–1 mm) remained around the tube (Fig. 5a). Once prepared, the sample was submerged in degassed 10 mM KCl solution and sealed in a Scholander pressure chamber such that the metal tube protruded through the top. The tube was then filled with water through the open end and connected via flexible tubing to a balance that recorded the flow rate. The expected path of water is marked by dark blue arrows in Fig. 5a, with the expectation that significant flow would occur via a single layer of bordered pits.

Determination of the pressure–flow relationship for gymnosperms was performed on five twigs per species. To ensure that the majority of water flowed via a single layer of bordered pits while limiting the number of tracheids open at both ends, twigs were cut under water to a length equal to 1.2 times the average tracheid length for that sample. Samples were then forced into the metal tube such that the xylem completely sealed the tube (Fig. 5b). Once the sample was mounted in the tube, the same procedure as for vessel-bearing species was followed (see flow path in Fig. 5b, marked by dark blue arrows).

Applied pressure across the samples ranged from ~0.03 MPa to ~2.0 MPa in approximate steps (0.03, 0.05, 0.1, 0.2, 0.4, 0.8, 1.0, 1.5, and 2.0 MPa). Flow rate was measured after steady-state conditions were observed for both pressure and flow.

### Reporting summary

Further information on research design is available in the Nature Portfolio Reporting Summary linked to this article.

## Supplementary information

Transparent Peer Review file

Supplementary Information

Description of Additional Supplementary files

Supplementary Data

Reporting Summary

## Data Availability

All data supporting the findings of this study are available within the paper and its Supplementary Information. The numerical source data for the graphs can be found in the Supplementary Data.

## References

[1] Tyree, M. T. & Zimmermann, M. H. *Xylem Structure and the Ascent of Sap* (Springer Science & Business Media, 2013).

[2] Pittermann, J., Sperry, J. S., Hacke, U. G., Wheeler, J. K. & Sikkema, E. H. Torus-margo pits help conifers compete with angiosperms. *Science* **310**, 1924 (2005). 16373568 10.1126/science.1120479

[3] Choat, B., Cobb, A. R. & Jansen, S. Structure and function of bordered pits: new discoveries and impacts on whole-plant hydraulic function. *N. Phytol.* **177**, 608–626 (2008). 10.1111/j.1469-8137.2007.02317.x 18086228

[4] MacDuffie, R. C. Vessels of the gnetalean type in angiosperms. *Bot. Gaz.* **71**, 438–445 (1921).

[5] Thompson, W. P. The anatomy and relationships of the Gnetales I. The genus Ephedra1. *Ann. Bot.* **os-26**, 1077–1104 (1912).

[6] Carlquist, S. Wood, bark, and stem anatomy of Gnetales: a summary. *Int. J. Plant Sci.* **157**, S58–S76 (1996).

[7] Dute, R. R. et al. Pit membranes of Ephedra resemble gymnosperms more than angiosperms. *IAWA J.* **35**, 217–235 (2014).

[8] Jacobsen, A. L. Diversity in conduit and pit structure among extant gymnosperm taxa. *Am. J. Bot.* **108**, 559–570 (2021). 33861866 10.1002/ajb2.1641

[9] Purusatama, B. D. et al. Qualitative anatomical characteristics of compression wood, lateral wood, and opposite wood in a stem of ginkgo biloba L. *J. Korean Wood Sci. Technol.* **46**, 125–131 (2018).

[10] Sperry, J. S., Adler, F. R., Campbell, G. S. & Comstock, J. P. Limitation of plant water use by rhizosphere and xylem conductance: results from a model. *Plant Cell Environ.* **21**, 347–359 (1998).

[11] Choat, B., Brodie, T. W., Cobb, A. R., Zwieniecki, M. A. & Holbrook, N. M. Direct measurements of intervessel pit membrane hydraulic resistance in two angiosperm tree species. *Am. J. Bot.* **93**, 993–1000 (2006). 21642164 10.3732/ajb.93.7.993

[12] Choat, B., Jansen, S., Zwieniecki, M. A., Smets, E. & Holbrook, N. M. Changes in pit membrane porosity due to deflection and stretching: the role of vestured pits. *J. Exp. Bot.* **55**, 1569–1575 (2004). 15181107 10.1093/jxb/erh173

[13] Park, K. et al. Viscous flow in a soft valve. *J. Fluid Mech.* **836**, R3 10.1017/jfm.2017.805 (2018).

[14] Schulte, P. J. & Hacke, U. G. Solid mechanics of the torus-margo in conifer intertracheid bordered pits. *N. Phytol.* **229**, 1431–1439 (2021). 10.1111/nph.16949 32981122

[15] Domec, J.-C., Lachenbruch, B. & Meinzer, F. C. Bordered pit structure and function determine spatial patterns of air-seeding thresholds in xylem of Douglas-fir (Pseudotsuga menziesii; Pinaceae) trees. *Am. J. Bot.* **93**, 1588–1600 (2006). 21642104 10.3732/ajb.93.11.1588

[16] Sperry, J. S. & Tyree, M. T. Water-stress-induced xylem embolism in three species of conifers. *Plant Cell Environ.* **13**, 427–436 (1990).

[17] Beikircher, B., Ameglio, T., Cochard, H. & Mayr, S. Limitation of the Cavitron technique by conifer pit aspiration. *J. Exp. Bot.* **61**, 3385–3393 (2010). 20551085 10.1093/jxb/erq159

[18] Choat, B. et al. Global convergence in the vulnerability of forests to drought. *Nature* **491**, 752–755 (2012). 23172141 10.1038/nature11688

[19] Pittermann, J., Sperry, J. S., Hacke, U. G., Wheeler, J. K. & Sikkema, E. H. Inter-tracheid pitting and the hydraulic efficiency of conifer wood: the role of tracheid allometry and cavitation protection. *Am. J. Bot.* **93**, 1265–1273 (2006). 21642190 10.3732/ajb.93.9.1265

[20] Hacke, U. G., Sperry, J. S., Wheeler, J. K. & Castro, L. Scaling of angiosperm xylem structure with safety and efficiency. *Tree Physiol.* **26**, 689–701 (2006). 16510385 10.1093/treephys/26.6.689

[21] Song, Y., Poorter, L., Horsting, A., Delzon, S. & Sterck, F. Pit and tracheid anatomy explain hydraulic safety but not hydraulic efficiency of 28 conifer species. *J. Exp. Bot.* **73**, 1033–1048 (2022). 34626106 10.1093/jxb/erab449 PMC8793876

[22] Zelinka, S. L. et al. Force-displacement measurements of earlywood bordered pits using a mesomechanical tester. *Plant Cell Environ.* **38**, 2088–2097 (2015). 25754548 10.1111/pce.12532

[23] Vincent, O., Sessoms, D. A., Huber, E. J., Guioth, J. & Stroock, A. D. Drying by cavitation and poroelastic relaxations in porous media with macroscopic pores connected by nanoscale throats. *Phys. Rev. Lett.* **113**, 134501 (2014). 25302891 10.1103/PhysRevLett.113.134501

[24] Stroock, A. D., Pagay, V. V., Zwieniecki, M. A. & Holbrook, N. M. The Physicochemical Hydrodynamics of Vascular Plants. *Annu. Rev. Fluid Mech.* **46**, 615–642 (2014).

[25] Gibson, L. J. The hierarchical structure and mechanics of plant materials. *J. R. Soc. Interface* **9**, 2749–2766 (2012). 22874093 10.1098/rsif.2012.0341 PMC3479918

[26] Zwieniecki, M. A., Melcher, P. J. & Holbrook, N. M. Hydraulic properties of individual xylem vessels of Fraxinus americana. *J. Exp. Bot.* **52**, 257–264 (2001). 11283170

[27] Zanne, A. E., Sweeney, K., Sharma, M. & Orians, C. M. Patterns and consequences of differential vascular sectoriality in 18 temperate tree and shrub species. *Funct. Ecol.* **20**, 200–206 (2006).

[28] Jansen, S. et al. Intervascular pit membranes with a torus in the wood of Ulmus (Ulmaceae) and related genera. *N. Phytol.* **163**, 51–59 (2004). 10.1111/j.1469-8137.2004.01097.x 33873781

[29] Park, K. et al. Fluid-structure interactions enable passive flow control in real and biomimetic plants. *Phys. Rev. Fluids* **6**, 123102 (2021).

[30] Jensen, K. H. et al. Sap flow and sugar transport in plants. *Rev. Mod. Phys.* **88**, 035007 (2016).

