# Supplementary material for: Torus-margo pits cannot function in vessel-bearing angiosperms
Source: Commun Biol. 2026 Apr 6;9:1026. doi: 10.1038/s42003-026-09800-x (PMC13421541; doi:10.1038/s42003-026-09800-x)
Supplement: Supplementary file 2 — Supplementary Information [file 42003_2026_9800_MOESM2_ESM.pdf]

Supplemental Information.

Supplemental Table 1. Anatomical data set used in the study.

**Angiosperm species** Citation – vessel L	**Vessel/tracheid length (m)**		**Vessel / tracheid diameter (mm)**	**Tree height (m)**	**P50 (MPa)**[4,5] (average value for the group)
	mean	maximum			
Acacia amoena[6]	0.04	0.12		3	(2.936)
Acacia greggii[7]	0.0476	0.28	39.7	15	0.88
Acalypha wilkesiana[8]		0.52		3	(2.936)
Acer grandidentatum[2,9]	0.032	0.032	25.45	12.5	(2.936)
Acer negundo[9,10]	0.0333	0.3	35.19	25	1.34
Acer palmatum[11]	0.03			10	1.88
Acer rubrum[12–14]	0.0312	0.16	45.33	38	1.97
Acer saccharinum[12,15]	0.033	0.4	33.3	35	3.87
Adenostoma fasciculatum[15,16]	0.0895	0.09	24.13	3.5	7.98
Adenostoma sparsifolium[16]	0.48	0.48	27.61	2	4.89
Alnus cordata[17]	0.0344	0.12	48	25	1.4
Alnus crispa[18]	0.0095	0.05	19	12	1.71
Alnus incana[18]	0.0097	0.035	22	20	1.7
Ambrosia dumosa[15]	0.091	0.091	28.54	0.9	1.05
Amelanchier alnifolia[19]	0.0555			8	4.37
Amelanchier utahensis[19]	0.0284			4	6.55
Anemopaegma puberulum[20]	0.26	1.15	50	30	(2.936)
Annona glabra[21]	0.0554		64.6	12	3.33
Antigonon leptopus[20]	0.86	0.86	27	7	(2.936)
Asparagus falcatus[20]		2.11	20	7	(2.936)
Arctostaphylos glandulosa[15,16]	0.0507	0.051	26.23	2.5	4.41
Arctostaphylos glauca[16]	0.3	0.3	26.6	6	7.29
Arctostaphylos patula[2,9]	0.0786	0.079	24.69	2	(2.936)
Argyreia nervosa[20]	0.57	1.31	64	10	(2.936)
Aristolochia gigantea[20]		1.71	23	5	(2.936)
Aristolochia maxima[20]		1.89	116	5	(2.936)
Aristolochia veraguensis[20]		2.6	108	3	(2.936)
Arrabidaea corallina[20]	0.69	2.5	32	3	(2.936)
Arrabidaea podopogon[20]	0.61	1.54	29.5	5	(2.936)
Artaboytrys hexapetalus[20]	0.05	0.27	34	2	(2.936)
Artemisia californica[15]	0.1011	0.101	29.09	2.5	1.83
Artemisia tridentata[22]	0.0265	0.0859	24	3	4.9

Atriplex acanthocarpa[7]	0.0294	0.14	23.4	1.2	(2.936)
Atriplex canescens[7,15]	0.0292	0.1	24.74	1.2	5.71
Atriplex polycarpa[7]	0.0149	0.06	19.8	2	1.98
Atriplex semibaccata[7]	0.0246	0.1	19.8	2	(2.936)
Barringtonia racemosa[23]	0.1391	0.45	30	8	(2.936)
Bauhinia aculeata[24]	0.0419	0.47	29	10	(2.936)
Bauhinia blakeana[20]	0.23	1	52	6	(2.936)
Bauhinia corymbosa[20]	0.1	1.84	23	8	(2.936)
Bauhinia fossoglensis[24]	0.1602	0.73	41	8	(2.936)
Bauhinia galpinii[24]	0.0864	1.84	40.33	3.5	(2.936)
Bauhinia purpurea[24]	0.1272	0.77	38.25	5.2	(2.936)
Bauhinia tenuiflora [8]		3.5		12	(2.936)
Bauhinia vahlii[20]	0.2667	1.17	39.67	30	(2.936)
Bauhinia variegata[20]	0.11	1.01	48	12	(2.936)
Betula occidentalis[18,25]	0.0326	0.25	27	14	1.52
Betula papyrifera[18,25]	0.0185		34	20	2.34
Betula polulifolia[12–14]		0.55		9	(2.936)
Bougainvillea spectabilis[20]	0.38	0.43	27	12.2	(2.936)
Calligonum aphyllum[7]	0.0328	0.14	37.1	1.2	(2.936)
Camarostaphylis diversifolia[11]	0.27	0.27	21.93	8.8	(2.936)
Carya glabra[2,9]	0.3312	0.331	70.38	40	2.1
Ceanothus crassifolius[2,16]	0.0423	0.042	25.45	3	9.4
Ceanothus cuneatus[11,12]	0.0538	0.054	22.12	3.5	7.96
Ceanothus leucodermis[11]	0.45	0.45	25.62	4	3.56
Ceanothus megacarpus[11,12]	0.067	0.067	26.05	3	8.08
Ceanothus oliganthus[11,12]	0.0566	0.057	26.19	3	4.13
Ceanothus spinosus[11,12]	0.0555	0.056	26.84	3	4.68
Ceanothus velutinus [2]	0.0583	0.058	29.21	3	3.63
Celtis australis [26]	0.04			25	(2.936)
Cercocarpus betuloides [16]	1.04	1.04	33.94	9.1	7.46
Cercocarpus ledifolius [19]	0.0189			11	4.96
Cercocarpus montanus [19]	0.0394			6.1	5.58
Cercosarpus intrictusself	0.02	0.03		10	(2.936)
Cinnamomum camphora[23]	0.1184	0.25	19.5	30	1.24
Citrus limonia[27]	0.19	0.45		10	(2.936)
Coleogyne ramosissima[15]	0.0587	0.059	18.6	1.2	(2.936)
Combretum latifolium[8]		3		30	(2.936)

Combretum paniculatum[20]	1.85	1.99	49	12	(2.936)
Dalbergia brownei[20]	0.12	0.55	48	5	(2.936)
Daphne spp (self)	0.02	0.08		4	3.35
Derris scandens[20]	0.37	1.5	14	15	(2.936)
Eriogonum cinereum[15]	0.087	0.087	32.06	1.2	1.83
Eucalyptus camaldulansis[27]	0.28	0.75		45	(2.936)
Eugenia uniflora[27]	0.28	0.75		8	(2.936)
Fagus grandifolia[12–14]		0.5		35	(2.936)
Fagus sylvatica f. purpurea[28]	0.0681		23.4	18	3.2
Flaveria brownii[29]	0.0239	0.12	22.7	0.7	(2.936)
Flaveria chloraefolia[29]	0.0194	0.1	27.3	0.7	(2.936)
Flaveria pringlei[29]	0.0448	0.14	43.5	0.7	(2.936)
Flaveria robusta[29]	0.0416	0.14	38.7	0.7	(2.936)
Flaveria sonorensis[29]	0.0523	0.2	26.2	0.7	(2.936)
Flaveria vaginata[29]	0.0252	0.1	25.5	0.7	(2.936)
Fraxinus americana[12]	0.7	7		40	1.92
Fraxinus pennsylvanica[2]	0.2526	0.253	39.65	45	0.9
Garrya veatchii[16]	0.35	0.35	28.4	2	6.02
Halimodendron halodendron[7]	0.0432	0.18	31.1	3	(2.936)
Haloxylon aphyllum[7]	0.0415	0.16	24.2	8	(2.936)
Hazardia squarrosa[15]	0.0459	0.046	32.21	2	1.33
Heteromeles arbutifolia[16]	0.86	0.86	26.41	5	2.43
Hippocratea volubilis[24]	0.4021	2.2	77.67	10	(2.936)
Hymenoclea salsola[15]	0.0587	0.059	28.65	2.5	(2.936)
Ilex verticulata[12–14]		0.25		5	(2.936)
Isomeris arborea[15]	0.0224	0.022	17.73	2	(2.936)
Lagerstroemia villosa[8]		1		15	(2.936)
Larrea tridentata[2,7,15]	0.1177	0.16	34.01	3	(2.936)
Lasiococca comberi [8]		0.23		10	(2.936)
Lepidospartum squamatum[15]	0.0684	0.068	33.5	1.5	(2.936)
Ligustrum lucidum[11]	0.36			10	(2.936)
Lotus scoparius [15]	0.0687	0.069	38.03	1.5	(2.936)
Luzuriaga marginata[20]		1.1	31	3	(2.936)
Macfadyena unguis-cati[20]	0.49	2.55	22.33	30	(2.936)
Malosma laurina[15,16]	0.1524	0.152	55.35	5	(2.936)
Malus domestica[30]	0.073	0.5		4.5	(2.936)
Mansoa allicea[20]	0.84	0.84	22	5	(2.936)
Mansosa verrucifera[20]		1.26	25	4	(2.936)
Mascagnia psilophylla[20]		1.22	29	15	(2.936)

Mesua ferrea [8]		1.15		15	(2.936)
Microcos paniculata [8]		0.3		12	(2.936)
Morus alba[2]	0.3714	0.371	57.73	20	0.46
Nitraria schoberi[7]	0.0389	0.14	29.5	2	(2.936)
Nothofagus fusca[31]		34		35	(2.936)
Nothofagus menziesii[31]		24		30	(2.936)
Nothofagus solandri[31]		0.24		27	(2.936)
Olea europea[27]	0.45	1		10	7.2
Osmanthus americans[self]	0.02	0.06		4	(2.936)
Osmanthus fragrans[11]	0.04			10	(2.936)
Oxydendron arboreum[2]	0.1689	0.169	30.39	20	3.17
Oxytenia acerosa[7]	0.0482	0.18	38.3	1.8	(2.936)
Pachystima myrsinites[2]	0.0364	0.036	16.89	1	(2.936)
Parthenocissus quinquefolia[32]	0.0959			30	(2.936)
Passiflora coccinea[24]	0.1416	1.79	25.67	3.6	(2.936)
Paulownia fortunei[11]	0.255			20	(2.936)
Peixotoa glabra[20]	0.23	1.68	27.5	10	(2.936)
Persea americana[27]	0.33	0.9		20	(2.936)
Pithecoctenium crucigerum[24]	0.5927	6.25	49.33	3	(2.936)
Platanus acerifolia[11]	0.18			30	(2.936)
Populus balsamifera [28,33]	0.0703	0.21	33.6	40	1.8
Populus tremuloides[12–14]	0.0191	0.25	23	18	2.74
Prosopis glandulosa[7]	0.056	0.32	44.2	9	1.57
Prunus ilicifolia[16]	0.22	0.22	26.78	15	4.39
Prunus persica[34]	0.1701		86	7	5.17
Pueraria montana[2]	0.6955	0.696	149.48	20	0.6
Purshia tridentata[19]	0.0391			30	4.3
Pyrostegia venusta[20]	1.53	1.53	39	5	(2.936)
Quercus agrifolia[16]	1.96	1.96	50.88	25	1.97
Quercus alba (early wood)[35]	0.1153	1.3	79.9	30	1.37
Quercus berberidifolia[15,16]	0.1779	0.178	46.83	2	0.26
Quercus gambelii[2,25]	0.1656	0.35	37.62	9	0.18
Quercus prinus[2]	0.1571	0.157	48.37	40	1.7
Quercus rubra[12]	0.4	10		28	1.61
Quercus rubra (early wood)[35]	0.1159	1.6	79.7	28	1.61
Quercus wislizenii[16]	1.45	1.45	38.14	22	2.49
Rhamnus californica[16]	1.06	1.06	29.56	3.6	2.51
Rhamnus crocea[16]	1.83	1.83	23.66	2	5.17
Rhamnus ilicifolia[16]	1.23	1.23	26.15	4	5.92

Rhododendron maximum[36]	0.0246		21.5	4	2.2
Rhus integrifolia[16]	1.16	1.16	35.43	8	1.8
Rhus ovata[15,16]	0.2216	0.222	42.26	10	1.25
Rhus trilobata[15,16]	0.0997	0.1	38.86	2	2.95
Robinia pseudoacacia[11]	0.18			40	0.5
Robinia pseudoaccacia[37]	0.196			60	0.5
Salix exigua[10]	0.0457	0.15	30.15	7	1.3
Salix pulchra[38]	0.1081	0.3	24	4.6	1.5
Salix viminalis[39]	0.0526			6	1.5
Salvia leucophylla [15]	0.0814	0.081	38.47	1.5	(2.936)
Salvia mellifera[15]	0.0668	0.067	30.78	2	(2.936)
Sambucus cerulea[10]	0.0449	0.3	37.59	9	1.43
Saritaea magnifica[24]	0.1346	2.7	45.33	10	(2.936)
Schinus mole[2]	0.02	0.07		7	1.68
Serjania polyphylla[20]	0.75	2.2	25	10	(2.936)
Sorbus scopulina[19]	0.0477		15	3.8	2.77
Spartium junceum[40]	0.0573	0.057		4	(2.936)
Stigmaphyllon periplocifolium[20]		1.51	23	4	(2.936)
Styphnolobium japonicum[11]	0.79			15	(2.936)
Syzygium cumini[8]		0.7		20	(2.936)
Tecomaria capensis[23]	0.0506	0.15	12.7	3	(2.936)
Thunbergia grandiflora[20]	1.28	1.7	42	2.5	(2.936)
Toxicodendron diversilobum[41]	0.0565	0.4	92	30	(2.936)
Toxicodendron diversilobum[41]	0.0782	0.25	92	30	(2.936)
Trichilia dregeana[23]	0.0896	0.25	28	25	2.66
Ulmus americana[42]	0.032			30	(2.936)
Umbellularia californica[16]	0.9	0.9	52.04	30	1.35
Vaccinium corymbosum[12–14]		1.25		4	(2.936)
Viburnum cassinoides[12–14]		1.5		5	2.25
Vitis labrusca [12]	1.8	8		10	(2.936)
Vitis labrusca/riparia[43]	0.416	1.4	200	5	0.15
Vitis rotundifolia[24]	0.3217	3.8	96	3	0.15
Vitis vinifera[10,19,44]	0.1503	1	69.18	4	0.75
**Torus-margo bearing gymnosperms**					
Abies balsamea[45]	0.00336		35.4	20	3.87
Abies concolor[45]	0.00351		40.8	60	3.74
Abies lasiocarpa [1]	0.00193756			50	7.12
Agathis australis [1]	0.00149914		17	40	2.58

Agathis ovata [1]	0.0019139		22	25	1.77
Araucaria laubenfelsii [1]	0.00144891		25	40	2.24
Callitropsis nootkatensis[45]	0.00232		28.2	40	(4.958)
Calocedrus decurrens[45]	0.00297		34.2	40	7.75
Chamaeciparis arizonica[45]	0.0017		23.2	25	5.15
Cryptomeria japonica[46]	0.0023		24	70	4.55
Cunninghamia lanceolata[46]	0.00214			46	6.93
Cupressus glabra[45]	0.00181		22.4	5	10.8
Cupressus lusitanica[46]	0.00241			30	(4.958)
Cupressus sargentii[45]	0.00291		31.8	15	(4.958)
Dacrydium cupressinum[1]	0.00164981		20	35	3.08
Ginkgo giloba[self]	0.0025		35	20	4.62
Juniperus osteosperma[45]	0.00159		23.5	6	6.92
Juniperus scopulorum [1]	0.00078487		11	15	(4.958)
Juniperus virginiana[45]	0.00198		25.1	20	6.6
Larix laricina[45]	0.00354		42.1	20	(4.958)
Larix occidenlalis[45]	0.00337		44.4	60	4.31
Phyllocladus trichomanoides [1]	0.00167796		27	25	7.02
Picea engelmanni[45]	0.00289		36.6	40	4.91
Picea glauca[45]	0.00334		36.8	30	4.3
Picea mariana[45]	0.00311		32.5	15	5.93
Pinus aristata[45]	0.0018		26.6	6	(4.958)
Pinus banksiana[45]	0.00314		34.9	22	(4.958)
Pinus caribaea [1]	0.00222617		30	50	3.27
Pinus concorta[45]	0.0029		35.4	50	3.67
Pinus contorta[45]	0.00190432			50	3.67
Pinus edulis[45]	0.00227		32.3	6.1	4.88
Pinus elliottii[46]	0.0037			30	(4.958)
Pinus flexilis[45]	0.00237		32.6	20	3.71
Pinus glabra[47]	0.00314			30	(4.958)
Pinus lambertina[45]	0.00398		44.5	60	(4.958)
Pinus monophylla [1]	0.00098897		20	20	5.55
Pinus patula[48]	0.00237		26	30	(4.958)
Pinus ponderosa[45]	0.00337		35.6	50	2.67
Pinus resinosa[45]	0.00336		37.4	35	(4.958)
Pinus strobus[45]	0.00373		41.8	70	(4.958)

Pinus sylvestris[46]	0.0031		36	40	3.61
Pinus taeda[46]	0.00371			46	(4.958)
Podocarpus acutifolius[49]	0.0018		22	7	(4.958)
Podocarpus dacrydioides[49]	0.0034		30	36	(4.958)
Podocarpus ferrugineus[49]	0.0028		24	30	(4.958)
Podocarpus hallii[49]	0.0028		25	28	(4.958)
Podocarpus laetus[1]	0.00133925		23	25	(4.958)
Podocarpus nivalis[49]	0.0014		19	3	(4.958)
Podocarpus spicatus[49]	0.00328		30	29	(4.958)
Podocarpus totara[49]	0.0028		28	34	(4.958)
Prumnopitys ferruginea[1]	0.00162555		25	25	(4.958)
Pseudotsuga menziesii (coastal B.C.)[45]	0.00362		41.5	100	(4.958)
Pseudotsuga menziesii (interior B.C.)[45]	0.00314		38.1	80	(4.958)
Retrophyllum minor [1]	0.00172428		19	10	2.17
Sequoia sempervirens[45]	0.00412		43.7	120	6.75
Sequoiadendron giganteum[50]	0.005		67	100	6.64
Taxodium distichum [1]	0.00164024		41	40	2.14
Thuja occidentalis[45]	0.00259		28.6	61	3.57
Tsuga canadensis[45]	0.00363		38.8	31	3.07
Wollemia nobilis[51]	0.00474		33	40	(4.958)
**EPHEDRAS**	Tracheid length (m)	Internode length (m)	Conduit diameter (mm)	Height (m)	
E. americana[52,53]	0.000593	0.025	27	3.5	(4.958)
E. andina Poepp. ex Mey.[52,53]	0.000748	0.025	32	1.8	(4.958)
E. antisyphilitica[52,53]	0.000697	0.025	34	2	(4.958)
E. aspera Engelmann ex Wats[52,53]	0.000813	0.06	28	1	(4.958)
E. boelckei Roig[52,53]	0.000785	0.03	41	5	(4.958)
E. boelckei[52,53]	0.000941	0.03	49	5	(4.958)
E. breana Phil.[52,53]	0.000473	0.03	21	1.2	(4.958)
E. californica Watson[52,53]	0.000746	0.025	39	1.2	(4.958)
E. clokeyi Cutler[52,53]	0.000807	0.05	29	1	(4.958)
E. compacta Rose[52,53]	0.000493	0.02	23	1	(4.958)
E. fasciculata A. Nelson[52,53]	0.000693	0.05	35	1	(4.958)
E. funerea Coville & Morton[52,53]	0.000722	0.02	32	1.2	(4.958)
E. multiflora Phil. ex Stapf[52,53]	0.000517	0.03	23	1.5	(4.958)
E. nevadensis Watson[52,53]	0.000556	0.025	33	1.2	(4.958)
E. ochreata Miers[52,53]	0.000434	0.03	26	1	(4.958)

E. pedunculata[52,53]	0.001006	0.07	43	7	(4.958)
E. rupestris Bentham[52,53]	0.000399	0.01	21	1.2	(4.958)
E. torreyana Watson[52,53]	0.000649	0.04	35	1	(4.958)
E. triandra Tul[52,53]	0.000595	0.04	22	2	(4.958)
E. trifurca[52,53]	0.000503	0.1	40	5	(4.958)
E. tweediana Fisch. & Mey.[52,53]	0.000553	0.05	26	2	(4.958)
E. viridis Coville[52,53]	0.000677	0.03	43	1	(4.958)
E. alata Decaisne[52,53]	0.000755	0.05	35	0.8	(4.958)
E. altissima Desf.[52,53]	0.001167	0.02	44	2	(4.958)
E. aphylla Forssk.[52,53]	0.000502	0.05	31	1	(4.958)
E. campylopoda C. A. Mey.[52,53]	0.000606	0.03	24	5	(4.958)
E. ciliata C. A. Mey[52,53]	0.000562	0.02	26	0.8	(4.958)
E. distachya L. var. distachya[52,53]	0.000683	0.03	27	1	(4.958)
E. equisetina Bunge[52,53]	0.000627	0.015	38	1.5	(4.958)
E. foliata Boiss.[52,53]	0.000724	0.02	42	2	(4.958)
E. fragilis Desf.[52,53]	0.000979	0.03	49	1.8	(4.958)
E. gerardiana Wall.[52,53]	0.000515	0.01	24	1	(4.958)
E. intermedia Schrenk[52,53]	0.000578	0.03	35	1	(4.958)
E. kokanica Regel[52,53]	0.000491	0.02	36	1	(4.958)
E. lomatolepis Schrenk[52,53]	0.000593	0.05	30	1	(4.958)
E. major Host[52,53]	0.000566	0.02	34	2	(4.958)

1. Pittermann, J., Sperry, J. S., Hacke, U. G., Wheeler, J. K. & Sikkema, E. H. Inter-tracheid pitting and the hydraulic efficiency of conifer wood: the role of tracheid allometry and cavitation protection. *Am. J. Bot.* **93**, 1265–1273 (2006).
2. Hacke, U. G., Sperry, J. S., Wheeler, J. K. & Castro, L. Scaling of angiosperm xylem structure with safety and efficiency. *Tree Physiol.* **26**, 689–701 (2006).
3. Song, Y., Poorter, L., Horsting, A., Delzon, S. & Sterck, F. Pit and tracheid anatomy explain hydraulic safety but not hydraulic efficiency of 28 conifer species. *J. Exp. Bot.* **73**, 1033–1048 (2022).
4. Hacke, U. G., Spicer, R., Schreiber, S. G. & Plavcová, L. An ecophysiological and developmental perspective on variation in vessel diameter. *Plant Cell Environ.* **40**, 831–845 (2017).
5. Choat, B. *et al.* Global convergence in the vulnerability of forests to drought. *Nature* **491**, 752–755 (2012).
6. Williamson, V. G. & Milburn, J. A. Xylem vessel length and distribution: does analysis method matter? A study using Acacia. *Aust. J. Bot.* **65**, 292 (2017).
7. Kocacinar, F. & Sage, R. F. Photosynthetic pathway alters hydraulic structure and function in woody plants. *Oecologia* **139**, 214–223 (2004).
8. Gao, H. *et al.* Vessel-length determination using silicone and air injection: are there artifacts? *Tree Physiol.* **39**, 1783–1791 (2019).
9. Lens, F. *et al.* Testing hypotheses that link wood anatomy to cavitation resistance and hydraulic conductivity in the genus Acer. *New Phytol.* **190**, 709–723 (2011).
10. Sperry, J. S., Hacke, U. G. & Wheeler, J. K. Comparative analysis of end wall resistivity in xylem conduits. *Plant Cell Environ.* **28**, 456–465 (2005).
11. Peng, G. *et al.* The theory behind vessel length determination using gas flow rates and comparison between two pneumatic methods based on seven woody species. *J. Exp. Bot.* **73**, 5612–5624 (2022).

12. Zimmermann, M. H. & Jeje, A. A. Vessel-length distribution in stems of some American woody plants. *Can. J. Bot.* **59**, 1882–1892 (1981).
13. Zimmermann, M. H. & Potter, D. Vessel-length distribution in branches, stem and roots of Acer rubrum L. *IAWA J.* **3**, 103–109 (1982).
14. Aloni, R. & Zimmermann, M. H. Length, width, and pattern of regenerative vessels along strips of vascular tissue. *Bot. Gaz.* **145**, 50–54 (1984).
15. Hacke, U. G., Jacobsen, A. L. & Pratt, R. B. Xylem function of arid-land shrubs from California, USA: an ecological and evolutionary analysis. *Plant Cell Environ.* **32**, 1324–1333 (2009).
16. Jacobsen, A. L. *et al.* Xylem density, biomechanics and anatomical traits correlate with water stress in 17 evergreen shrub species of the Mediterranean-type climate region of South Africa. *J. Ecol.* **95**, 171–183 (2007).
17. Tognetti, R. & Borghetti, M. Formation and seasonal occurrence of xylem embolism in Alnus cordata. *Tree Physiol.* **14**, 241–250 (1994).
18. Sperry, J. S., Nichols, K. L., Sullivan, J. E. M. & Eastlack, S. E. Xylem embolism in ring-porous, diffuse-porous, and coniferous trees of northern Utah and interior Alaska. *Ecology* **75**, 1736–1752 (1994).
19. Wheeler, J. K., Sperry, J. S., Hacke, U. G. & Hoang, N. Inter-vessel pitting and cavitation in woody Rosaceae and other vesselled plants: a basis for a safety versus efficiency trade-off in xylem transport. *Plant Cell Environ.* **28**, 800–812 (2005).
20. Ewers, F. W., Fisher, J. B. & Chiu, S.-T. A survey of vessel dimensions in stems of tropical lianas and other growth forms. *Oecologia* **84**, 544–552 (1990).
21. Zotz, G., Tyree, M. T. & Patiño, S. Hydraulic architecture and water relations of a flood-tolerant tropical tree, Annona glabra. *Tree Physiol.* **17**, 359–365 (1997).
22. Kolb & Sperry. Transport constraints on water use by the Great Basin shrub,Artemisia tridentata. *Plant Cell Environ.* **22**, 925–935 (1999).
23. Vander Willigen, C., Sherwin, H. W. & Pammenter, N. W. Xylem hydraulic characteristics of subtropical trees from contrasting habitats grown under identical environmental conditions. *New Phytol.* **145**, 51–59 (2000).
24. Ewers, F. W. & Fisher, J. B. Techniques for measuring vessel lengths and diameters in stems of woody plants. *Am. J. Bot.* **76**, 645–656 (1989).
25. Sperry, J. S. & Sullivan, J. E. Xylem embolism in response to freeze-thaw cycles and water stress in ring-porous, diffuse-porous, and conifer species. *Plant Physiol.* **100**, 605–613 (1992).
26. Wheeler, E. A., LaPasha, C. A. & Miller, R. B. Wood anatomy of elm (Ulmus) and Hackberry (Celtis) species native to the United States. *IAWA J.* **10**, 5–26 (1989).
27. Pereira, L. *et al.* A semi-automated method for measuring xylem vessel length distribution. *bioRxiv* (2020) doi:10.1101/2020.08.04.234575.
28. Hacke, U. & Sauter, J. J. Vulnerability of xylem to embolism in relation to leaf water potential and stomatal conductance inFagus sylvaticaf.purpureaandPopulus balsamifera. *J. Exp. Bot.* **46**, 1177–1183 (1995).
29. Kocacinar, F., McKown, A. D., Sage, T. L. & Sage, R. F. Photosynthetic pathway influences xylem structure and function in Flaveria (Asteraceae). *Plant Cell Environ.* **31**, 1363–1376 (2008).
30. Cohen, S., Bennink, J. & Tyree, M. Air method measurements of apple vessel length distributions with improved apparatus and theory. *J. Exp. Bot.* **54**, 1889–1897 (2003).
31. Middleton, T. M. & Butterfield, B. G. Vessel length distribution in the stems of three New Zealand species of Nothofagus. *Wood Sci. Technol.* **24**, (1990).
32. McElrone, A. J., Sherald, J. L. & Forseth, I. N. Interactive effects of water stress and xylem-limited bacterial infection on the water relations of a host vine. *J. Exp. Bot.* **54**, 419–430 (2003).
33. Hacke, U. & Sauter, J. J. Drought-Induced Xylem Dysfunction in Petioles, Branches, and Roots of Populus balsamifera L. and Alnus glutinosa (L.) Gaertn. *Plant Physiol.* **111**, 413–417 (1996).
34. Vercambre, G., Doussan, C., Pages, L., Habib, R. & Pierret, A. Influence of xylem development on axial hydraulic conductance within Prunus root systems. *Trees (Berl. West)* **16**, 479–487 (2002).
35. Cochard, H. & Tyree, M. T. Xylem dysfunction in Quercus: vessel sizes, tyloses, cavitation and seasonal changes in embolism. *Tree Physiol.* **6**, 393–407 (1990).
36. Lipp, C. C. & Nilsen, E. T. The impact of subcanopy light environment on the hydraulic vulnerability of Rhododendron maximum to freeze-thaw cycles and drought. *Plant Cell Environ.* **20**, 1264–1272 (1997).
37. Ling-Ling, Z. & Rui-Qing, W. Xylem Vessel Length Determination in Robinia pseudoacacia L. by Simplified Air Injection. *Plant Sci. J.* (2016) doi:10.11913/psj.2095-0837.2016.60920.

38. Gorsuch, D. M. & Oberbauer, S. F. Effects of mid-season frost and elevated growing season temperature on stomatal conductance and specific xylem conductivity of the arctic shrub, Salix pulchra. *Tree Physiol.* **22**, 1027–1034 (2002).
39. Salleo, S. *et al.* A method for inducing xylem emboli in situ: experiments with a field-grown tree. *Plant Cell Environ.* **15**, 491–497 (1992).
40. Gullo, M. A. L., Trifilò, P. & Raimondo, F. Hydraulic architecture and water relations ofSpartium junceumbranches affected by a mycoplasm disease. *Plant Cell Environ.* **23**, 1079–1088 (2000).
41. Gartner, B. L. Stem hydraulic properties of vines vs. shrubs of western poison oak, Toxicodendron diversilobum. *Oecologia* **87**, 180–189 (1991).
42. Newbanks, D. Evidence for xylem dysfunction by embolization in dutch elm disease. *Phytopathology* **73**, 1060 (1983).
43. Sperry, J. S., Holbrook, N. M., Zimmermann, M. H. & Tyree, M. T. Spring filling of xylem vessels in wild grapevine. *Plant Physiol.* **83**, 414–417 (1987).
44. Jacobsen, A. L., Pratt, R. B., Tobin, M. F., Hacke, U. G. & Ewers, F. W. A global analysis of xylem vessel length in woody plants. *Am. J. Bot.* **99**, 1583–1591 (2012).
45. Bannan, M. W. The length, tangential diameter, and length/width ratio of conifer tracheids. *Can. J. Bot.* **43**, 967–984 (1965).
46. Vivian, M. A., Silva, A. M. O., Modes, K. S., Dobner Júnior, M., Silva Júnior, F. G. Características da madeira de Cunninghamia lanceolata (Chinese fir). *Scientia Forestalis* **49**, (2021).
47. Vivian, M. A., Santos, J. R. S. dos, Segura, T. E. S., Silva Júnior, F. G. da & Brito, J. O. Caracterização do bagaço de cana-de-açúcar e suas potencialidades para geração de energia e polpa celulósica. *Madera Bosques* **28**, (2022).
48. Modes, K. S., Ludwig, R. L., Vivian, M. A. & Stolberg, J. Wood quality of Pinus patula Schltdl & Cham for the pulp production. *RevistaÁrvore* **43**, e430207 (2019).
49. Patel, R. N. Wood anatomy of podocarpaceae indigenous to New Zealand: 2.Podocarpus. *N. Z. J. Bot.* **5**, 307–321 (1967).
50. Lazzarin, M. *et al.* TRACHEID AND PIT ANATOMY VARY IN TANDEM IN A TALL SEQUOIADENDRON GIGANTEUM TREE. *IAWA J.* **37**, 172–185 (2016).
51. Heady, R. D., Banks, J. G. & Evans, P. D. WOOD ANATOMY OF WOLLEMI PINE (WOLLEMIA NOBILIS, ARAUCARIACEAE). *IAWA J.* **23**, 339–357 (2002).
52. Carlquist, S. Wood, bark, and pith anatomy of old world species of Ephedra and summary for the genus. *Aliso* **13**, 255–295 (1992).
53. Carlquist, S. Wood and bark anatomy of the new world species of Ephedra. *Aliso* **12**, 441–483 (1989).

Supplemental Table 2. Model input and sensitivity analysis. Best parameter estimates, minimum, and maximum values.

Symbol	Parameter	Estimate (angiosperm)	Estimate (conifer)	Minimum	Maximum	Source
p_x	Xylem tension	-2.936 MPa	-4.958 MPa	*-2 MPa*	*-5 MPa*	(*19*)
K	Bulk modulus	5 GPa	5 GPa	*2 GPa*	*10 GPa*	(*26*)
$\Delta p_{\min}$	Torus-margo actuation stress	0.25 or 0.75 MPa	0.25 or 0.75 MPa	*0.1 MPa*	*1.0 MPa*	Fig. 1c

Supplemental Table 3. Model output and sensitivity analysis. Predict critical conduit length to plant height ratio ($l_{\max}/L$) based on the best estimate scenario. For the 8 combinations based on the minimum-to-maximum ranges (Supplemental Table 2), the mean, standard error of the mean, and the minimum and maximum values of $l_{\max}/L$ are given.

Symbol	Parameter	Estimate (angiosperms) (conifers)	Mean	Standard error of the mean	Minimum	Maximum
$l_{\max}/L$	Length ratio	5.3×10^{-4}<br>9.5×10^{-4}	8.8×10^{-4}	3.1×10^{-4}	1.0×10^{-4}	2.4×10^{-3}
