# Supplementary material for: Torus-margo pits cannot function in vessel-bearing angiosperms
Source: Commun Biol. 2026 Apr 6;9:1026. doi: 10.1038/s42003-026-09800-x (PMC13421541; doi:10.1038/s42003-026-09800-x)
Supplement: Supplementary file 3 — Description of Additional Supplementary files [file 42003_2026_9800_MOESM3_ESM.pdf]

Description of Additional Supplementary files

File name: Supplementary Data

Description: The numerical source data for the graphs
