# Supplementary material for: Torus-margo pits cannot function in vessel-bearing angiosperms
Source: Commun Biol. 2026 Apr 6;9:1026. doi: 10.1038/s42003-026-09800-x (PMC13421541; doi:10.1038/s42003-026-09800-x)
Supplement: Supplementary file 1 — Transparent Peer Review file [file 42003_2026_9800_MOESM1_ESM.pdf]

nature portfolio

Peer Review File

Torus-margo pits cannot function in vessel-bearing angiosperms

Corresponding Author: Professor Maciej Zwieniecki

This file contains all reviewer reports in order by version, followed by all author rebuttals in order by version.

Attachments originally included by the reviewers as part of their assessment can be found at the end of this file.

Version 0:

Reviewer comments:

Reviewer #1

(Remarks to the Author)
In this report, Zwieniecki et al tried to understand the absence of torus-margo pit pores in vessel-bearing plants. They asked two important questions 1. How much force must be applied to close a torus-margo pit pore 2. Why are vessels unable to generate this stress? The authors articulated clear hypotheses and defined research objectives.

Here are some strengths of the paper. This study offers novel insights into the evolutionary divergence of hydraulic strategies between gymnosperms and angiosperms by focusing on the role of conduit length in influencing torus-margo pit functionality. Also, the study integrates anatomical, physiological, and theoretical approaches to address a complex question regarding embolism formation and pressure dynamics in plant xylem, which is impressive.

Major points
However, many areas need improvement before publication. The study relies on data from a relatively small number of species, which may not fully capture the diversity of hydraulic strategies within gymnosperms and angiosperms. I encourage the authors to analyze a few more species of angiosperms, at least n=3 is required.

The theoretical model proposed in the study makes simplifying assumptions about tissue properties and conduit dimensions, which fails to fully capture the complexity of plant physiology. Please refine the model to account for more realistic scenarios such as root length variation, genetic variation, and environmental conditions to improve its accuracy.

What are the underlying evolutionary processes driving the divergence of hydraulic strategies between conifers and vessel-bearing plants?

Minor points
In the main text, line 64, Fig. 2 is mentioned but the authors probably mean to cite Fig. 1.

Although data from 2 angiosperm and 4 gymnosperm species are shown in Figure 1, the names of only 5 of them are described in the method section.

Reviewer #2

(Remarks to the Author)
See separate pdf file

Reviewer #3

(Remarks to the Author)
Overall this is an interesting paper with a novel hypothesis suggesting the failure of angiosperms to utilize the torus-margo

pit membrane in the xylem of woody plants. There are a number of anatomical terminology issues throughout. The authors have also missed several key papers that address the same problem, answer some of the same questions, and either perform the same experiments (Sperry and Tyree 1990) or provide data for which the authors claim does not exist (Zelinka et al. 2015). These are not fatal omissions, and I believe that the inclusion of a discussion of those data (as well as the others cited below) will help to better contextualize the data presented in the manuscript. Most importantly the authors provide a compelling hypothesis, as well as evidence to support it. But, the Zelinka et al. 2015 severely undercuts one of the main claims of novelty and the authors will need to acknowledge it, as well as the data by Sperry and Tyree 1990 that provide estimates on the pressure required to close the torus-margo pit. What is novel here is the proposed mechanism. So, while some of the data presented here are not as novel as the authors have made them out to be, they are generally in line with existing data which can be used to further support their claims.

Specific comments:

The authors could improve the manuscript considerably by better articulating the conclusions section (the final paragraph before the methods, Lines 148-154). This isn't as strong as it could be. The text at times is not written for a broad audience and a significant knowledge of the plant hydraulics literature is required to fully follow the logic.

There are two problems with the abstract. While I appreciate the purpose of being concise and the constraints of the word limit, it isn't quite as simple as the authors have presented the problem. An important parallel thread in the evolution of hydraulic properties of plants is the ferns, which have tracheids but do not have torus-margo pit membranes. There are a few taxa that reportedly have 'vessels', such as Pteridium (Carlquist makes a note of this), but the fern example (tracheids with homogenous pit membranes) makes for a good comparison to the question addressed by this paper. There's a line (140) that briefly mentions the "dominance of conifers over lycopods and ferns", but there's no reference there and it is a bit misleading in terms of what the authors mean by 'dominance'. In terms of diversity the ferns outnumber the conifers, and lycophytes have been around for a much than conifers. It might be worth qualifying this statement (line 140) in terms of biomass or the evolutionary timescale that the authors are considering.

Second, there are examples of tori in angiosperms, such as in Ulmus and other related taxa (Ref 16). From the anatomical perspective the abstract, as stated, is true but there have been evolutionary attempts at something similar to the torus-margo pit membrane in angiosperms. The authors note this issue (Line 143), but it might be worth considering Coleman CM, Prather BL, Valente MJ, Dute RR & Miller ME. 2004. Torus lignification in hardwoods. IAWA J. 25: 435–447.

Lines 15-17. The authors should provide a reference for the "one-third of water vapor..." statement.

Line 20-21: It would be worth citing Brodribb et al. here since they show a direct connection between hydraulic failure and tissue death: https://doi.org/10.1111/nph.17577

Line 23: "that allow water, but not gas, to pass." This is only partially true based on our understanding of air-seeding and the movement of gas bubbles through the xylem. The pit membranes only prevent air movement up until a certain pressure threshold.

Lines 25-38: The anatomical terminology in this section is largely incorrect, or at least misleading. The authors have collapsed the pit and the pit membrane into a single unit for some reason, perhaps for simplicity? The first author is well-aware of the correct terminology so this is a curious choice. For example, in line 36 the authors use a mix of terminology to the detriment of their argument "torus-margo pits are 59 times more conductive than membranes". In the first part of this clause they combine the torus-margo pit membrane and the pit itself with this wording, and then only consider the homogenous pit membrane of the angiosperms in the second half of the clause. This is unnecessarily confusing. I think most importantly that the authors go through the manuscript and make careful edits and provide the correct terminology. This is critical because both the pit dimensions (aperture, dimensions of the internal chamber of the pit, etc.) have an influence over the resistance that can be considered separately from the membrane, whereas the authors are sometimes combing the chamber and the membrane, or apparently only considering the membrane in absence of the pit chamber. Paul Schulte's pit chamber and pit membrane modeling are worth considering as well, particularly given that he provides information about the resistance of the margo strand pores (https://doi.org/10.1111/nph.16949; https://doi.org/10.1111/j.1469-8137.2011.03986.x).

Line 41: Again, correct anatomical nomenclature is very important. Here, "suffice to bend the cellulose fibers by which the torus is suspended". The authors are talking about the margo strands. In the preceding sentence they use the term "margo gates", which is not an anatomical term. My recommendation is to use consistent terminology throughout.

Line 41-42: Zelinka et al 2015 measure force-displacement of torus-margo pit membranes (https://doi.org/10.1111/pce.12532). So, this sentence is not entirely accurate.

Line 42: "we note that vessels are longer". This should be corrected. In general, vessels are longer but there are species with long tracheids (e.g. Sequoia sempervirens) that are as long as some angiosperm vessels. The authors should also provide a reference here showing the relationship between conduit length and volume.

Line 43: "If an embolism occurs, the liquid volume occupied by the expanding gas is released into the surrounding tissue, which is under tension." This is stated as fact and unreferenced but I don't think there's any evidence supporting this idea. It is probably true based on current theory, but it is at this point unsubstantiated.

Line 46-47: "the air can migrate across the large gaps in the margo mesh" This statement is in conflict with line 23, see comment above.

Line 48: The authors should provide some rationale behind the <0.1 MPa value. Presumably this is based on Young-Laplace?

Line 49: The first question is answered by Zelinka et al. 2015, at least for Larix.

Line 52: "to point where the torus-margo can no longer function." This is vague. Function in what way? Do the authors mean that it is pushed against the pore opening and can no longer conduct water? This hypothesis needs to be better supported.

Lines 55-56: See Zelinka et al. 2015. The authors will need to put their work within the context of this paper, or sufficiently explain why the work presented here is sufficiently different.

Line 58: Again, Zelinka et al. 2015. Direct measurement of force:displacement of torus-margo pit membranes. Larix and Pinus.

Line 71: It would be interesting to perform this experiment in species with vestured pits, where the membrane is presumably supported and should not flex as much. See worth by Steven Jansen. E.g. Jansen, Steven, et al. "Vestured pits: do they promote safer water transport?." International Journal of Plant Sciences 164.3 (2003): 405-413.

Or, https://doi.org/10.1093/plphys/kiac223

In this second paper (see Fig 5) they model pore constriction size as a function or pressure, which should ultimately influence the conductivity measurements in the present manuscript.

Line 72: Again, the issue of terminology. "In tori bearing species". It really isn't the torus that is the important part, but the margo strands. The point at which the margo strands flex and allow the torus to seal the pore is the critical mechanism driving the results in Fig. 1. So, calling them 'tori bearing species' is a bit strange.

Line 74-75: "and we attribute it to the gradual occlusion of torus-margo pores." I don't understand why the authors would attribute the reduction in flow to 'occlusion'. The choice of wording here is problematic. Water is flowing through the pores between the margo strands, as well as through the pores on either side of the pit. At the point when the torus covers the pore, with some overlap in terms of the radius of the torus and the radius of the pore, the whole pit will not allow water to flow through. The water would have to move through the torus at this point, so whether the pores between the margo strands is occluded is not particularly relevant. Also, what would the 'torus-margo' pores be occluded by? Do the authors mean that the pores between the strands shrink?

Line 76: It would be interesting to compare the result here with that of Zelinka et al. 2015 "The force or calculated pressure needed to displace the pit membrane to an aspirated position was found to be less than 3 μN and is slightly lower than previous calculations of the pressure differences needed to cause aspiration".

Furthermore, Zelinka et al. 2015 note in their Discussion section that: "From hydraulic conductivity measurements, researchers have calculated an aspiration pressure difference of between 33 and 470 kPa (Gregory & Petty 1973; Sperry & Tyree 1990; Domec et al. 2006)."

Of particular relevance is the Sperry and Tyree 1990. Their Figure 12 shows essentially the same result as Fig. 1 here. Thus, the data in Fig. 1 are largely confirmatory given that Sperry and Tyree 1990 report changes in conductivity over essentially the same range of applied pressures as the authors do in this paper. They find the same results for two gymnosperms (Abies and Juniperus) and an angiosperm (Acer). Sperry and Tyree 1990's Acer data are more linear than what the authors find here in Fig. 1b, which is interesting as a point of comparison. It is essential that the authors cite this work and put the data into context.

Line 90 "that the primary function of pits is to limit the spread of gas pockets from one conduit to its neighbors." I would argue that this is primary function of the pit membrane, not the pit. I think this is probably just an issue with terminology.

Fig. 3. It would be worth noting in this figure where Welwitschia is situated on this gradient, as well as some of the vessel-less angiosperms, or even the ferns.

Line 147. If we look at vessel length distributions, see work by Tyree's group, or Zimmermann and Jeje 1981, we see that the longest vessels make up a very small proportion of the total vessels in a stem. Most of the vessels are skewed toward the shorter end of the spectrum. So, while you might find some exceptionally long vessels in ring-porous species (E.g. Quercus) or linanas with large diameter vessels (E.g. Vitis), those very long vessels (>1m in length) represent a small fraction of the total. The logic proposed here would suggest that there would be a strong selective pressure against torus-margo pit membranes because of the 'longest vessels' would be more vulnerable. This seems like a somewhat weak argument. What may be worth considering is that the ancestral state in the angiosperms is thought to be tracheid based xylem (Hacke et al. 2007; DOI: 10.1086/520724.) In this paper they propose many of the ideas in this present manuscript. "First, vesselless angiosperm wood should have much higher flow resistance than conifer wood because angiosperm tracheids lack low-

resistance torus-margo pits. Second, vesselless wood ought to be exceptionally safe from cavitation if the small cumulative area of pits between tracheids confers safety (the pit area hypothesis)."

Line 156: Taxon names should be in italics.

Version 2:

Reviewer comments:

Reviewer #1

(Remarks to the Author)
I reviewed this MS a very long ago. The authors addressed most of my concerns satisfactorily. The manuscript improved in readability, presentation quality and somewhat better clarity in the model. Tiny commen: in the abstract, in line 15, you may wish to remove "-" above the stop.

Reviewer #2

(Remarks to the Author)
See the document attached

Reviewer #3

(Remarks to the Author)
The authors have done a good job resolving the issues brought up by the reviewers and I have no major comments at this point. There are typos in the x-axis labels for Figs. 2 and 3 that should be corrected (Trachea should be Tracheid). I think the paper is now much easier to understand it provides an important contribution to our overall understanding of xylem evolution.

Version 3:

Reviewer comments:

Reviewer #2

(Remarks to the Author)
I am satisfied with the replies to my comments and suggestions. The revised version of this manuscript is much easier to read and provides convincing, novel evidence that torus-margo pits need to be associated with tracheids.

Open Access This Peer Review File is licensed under a Creative Commons Attribution 4.0 International License, which permits use, sharing, adaptation, distribution and reproduction in any medium or format, as long as you give appropriate credit to the original author(s) and the source, provide a link to the Creative Commons license, and indicate if changes were

made.
In cases where reviewers are anonymous, credit should be given to 'Anonymous Referee' and the source.
The images or other third party material in this Peer Review File are included in the article’s Creative Commons license, unless indicated otherwise in a credit line to the material. If material is not included in the article’s Creative Commons license and your intended use is not permitted by statutory regulation or exceeds the permitted use, you will need to obtain permission directly from the copyright holder.
To view a copy of this license, visit https://creativecommons.org/licenses/by/4.0/

Reviewers' comments:

Reviewer #1 (Remarks to the Author):

In this report, Zwieniecki et al tried to understand the absence of torus-margo pit pores in vessel-bearing plants. They asked two important questions 1. How much force must be applied to close a torus-margo pit pore 2. Why are vessels unable to generate this stress? The authors articulated clear hypotheses and defined research objectives.

Here are some strengths of the paper. This study offers novel insights into the evolutionary divergence of hydraulic strategies between gymnosperms and angiosperms by focusing on the role of conduit length in influencing torus-margo pit functionality. Also, the study integrates anatomical, physiological, and theoretical approaches to address a complex question regarding embolism formation and pressure dynamics in plant xylem, which is impressive.

Major points

However, many areas need improvement before publication. The study relies on data from a relatively small number of species, which may not fully capture the diversity of hydraulic strategies within gymnosperms and angiosperms. I encourage the authors to analyze a few more species of angiosperms, at least n=3 is required.

As requested, we have added an additional set of measurements for angiosperms. While it is true that the number of species included in the investigation of bordered pit responses to increasing pressure is small (a total seven species), each species was measured five times across a range of pressures. Thus, the total number of measurements is approximately 150. In addition, our findings are consistent with previous studies that used both modeling and experimental approaches, and we therefore feel that we have adequately captured the differences in response between torus-margo and homogeneous pit membranes.

The theoretical model proposed in the study makes simplifying assumptions about tissue properties and conduit dimensions, which fails to fully capture the complexity of plant physiology. Please refine the model to account for more realistic scenarios such as root length variation, genetic variation, and environmental conditions to improve its accuracy.

The important aspect of the model is its simplicity, which captures the key functional differences between torus-margo membranes and homogenous membranes separating adjacent water conduits. We also feel that the model effectively reflects the most relevant aspects of plants anatomical diversity that impose physical constraints on membrane functionality. However, to improve the clarity and perception of the model, we have added an analysis of the underlying principles governing the generation of the dynamic pressure acting on membranes during cavitation and embolus expansion events.

What are the underlying evolutionary processes driving the divergence of hydraulic strategies between conifers and vessel-bearing plants?

We expanded the introduction and discussed the only family of plants known to exhibit vessel-like structures coinciding with torus-margo membranes.

Minor points
In the main text, line 64, Fig. 2 is mentioned but the authors probably mean to cite Fig. 1.

Manuscript was entirely rewritten and checked for figure citation

Although data from 2 angiosperm and 4 gymnosperm species are shown in Figure 1, the names of only 5 of them are described in the method section.

Names are listed correctly now

Reviewer #2 (Remarks to the Author)

This paper explores how potential pressure reduction associated with embolism formation in conduits can be associated with two major pit membrane types in unicellular tracheids and multicellular vessels. Overall, it is interesting to test if the water released by embolism formation is causing any local pressure increase (i.e. xylem pressure becomes less negative), and how this may affect the valve effect of pit membranes to prevent embolism spreading. The torus-margo aspiration of conifers has been known for a long time, and closure of the pit aperture by a torus is likely an important mechanism for conifers to prevent embolism propagation, while different mechanisms are more likely in angiosperms.

We have expanded the results and discussion sections to include an analysis of velocity estimates within conduit elements undergoing embolism, as well as the generation of instantaneous dynamic pressure acting on torus-margo membranes across the range of conduit morphologies examined.

The experimental data suggest that torus-margo aspiration occurs at a pressure difference of 0.25 MPa (or 250 kPa). These values are not novel and largely in line with earlier papers, e.g. Gregory & Petty 1973, Sperry & Tyree 1990, Domec et al. 2006, Beikircher et al. 2010, Bouche et al. 2015 – based on hydraulic measurements, forces required for aspiration were between 8 to 36 μN, and the pressure difference to cause aspiration was between 33 to 470 kPa. A more direct estimation based on force displacement showed that only 3 μN was sufficient to aspirate a torus (Zelinka et al., 2015). Angiosperm pit membranes are clearly much stiffer, and may need a pressure difference above 2 MPa between neighbouring conduits for aspiration (Capron et al. 2014; Carmesin et al. 2023). I missa more balanced integration of earlier data nand literature references, although I fully agree that mechanical properties of pit membranes are not well known.

We expanded on our results to better integrate prior experimental and modeling estimates of torus-margo pit membrane pressure thresholds.

The expansion of an existing bubble inside a conduit is a more complicated topic than how this is presented here. The bubble size matters, and whether or not the Blake threshold has been reached or not. Bubbles can be stable in a liquid under negative pressure, and the compression pressure on a bubble is determined by the surface tension and the bubble radius. There is strong evidence for the presence of polar lipids in xylem sap, with a dynamic surface tension that can be very low or high. I am surprised to see that surfactants are ignored here as these have an effect on bubble stability, although this makes it hard to model the bubble behaviour and growth. The time of the bubble expansion, and thus the replacement of the liquid from an embolising conduit to a neighbouring conduit should also affect the intercellular pressure drop. Why is time not included in the model? Or is bubble expansion simply assumed to be very fast?

We have added an entire section on the velocity of bubble expansion within water-filled conduits. This analysis applies Washburn's 1921 original formula using available morphological data for both angiosperms and torus-margo bearing species. The effect of surfactants was not included in our analysis as their presence primarily reduce surface tension and thereby increase the rate of bubble expansion. While this would lead to a modest rise in dynamic pressure that could facilitate torus displacement in conifer-like anatomies, even a two to threefold increase in expansion velocity within the large vessels of angiosperms would still generate insufficient dynamic pressure to displace the torus.

The model is based on an axial, single pathway (Length L), while radial connectivity is not included. Conduits are not only connected axially, but also radially, so neighbouring conduits, but also fibres or

parenchyma cells may take up water released by embolised conduits. Why is any radial transport not considered in this model? This may raise questions about the l/L significance (Fig. 2 and 3).

By adding an analysis of the initial velocity of bubble expansion, we address both limits of the analytical approach. The most conservative scenario assumes that the expanding bubble enters an effectively unlimited tissue volume, such that pressure in the rest of the plant remains constant. In contrast, the most lenient scenario assumes that the absorbing volume is limited to a single conduit, as described in the pipe-line theory (Shinozaki K, Yoda K, Hozumi K, Kira T. 1964a. A quantitative analysis of plant form – the pipe model theory. I. Basic analyses. Japanese Journal of EcologyB: 97–105). This case allows for the maximum possible pressure buildup. Interestingly, both approaches yield similar results from opposite extremes: in the first case, angiosperms cannot generate sufficient dynamic pressure to displace the torus, whereas in the latter, they would experience high backlash pressures that likewise prevent torus aspiration. .

The abstract was rather unclear to me. Without reading the full paper, I did not get the main goal of the paper and the key message. It would be useful to mention that this paper is largely based on a model (Fig. 2), but does not really deal with the "physical conditions necessary for proper embolism isolation", which I found misleading.

We rewrote the abstract to adequately represent the findings.

Line 23: It is really not correct that xylem conduits (and pit membranes) are airtight because xylem sap is saturated or even oversaturated with gas. Gas molecules can also diffuse through cell wall pores, even if this process is somewhat slow.

We thank the reviewer for this important clarification. In the revised version, the entire introductory section has been rewritten. The new text now accurate describes the function of pit membranes as hydraulic partitions that isolate embolize conduits and limit the spread of air, without implying complete impermeability.

The terminology used for xylem anatomy is awkward. The term "pit pore", for instance, is used as a synonym for bordered pit, pit membrane, pit aperture, or a pit membrane pore. I would strongly recommend the authors to use the appropriate terms that are used for the various and geometrically complex openings in secondary cell walls of conduits.

We have rewritten and adjusted terminology in entire manuscript.

Line 83: Please explain more clearly why -3.4 MPa is taken as xylem pressure for conifers. Is this the average xylem pressure of many conifer species on an average day? This value seems rather negative, and is probably the pressure needed to create embolism. Please explain this more clearly.

We have adjusted the analysis to use average xylem tension P50 values for conifers and angiosperms based on Choat, B., Jansen, S., Brodribb, T. et al. Global convergence in the vulnerability of forests to drought. Nature 491, 752–755 (2012). https://doi.org/10.1038/nature11688) , where P_{50} = - 2.85 for angiosperms and P_{50} = -5.60 for gymnosperms. When available, species-specific P_{50} values were used.

Line 86 and 88: Where do these number come from? Any references, or is this a rough estimation about the number of tracheids/vessels in a single axial pathway of an average sized tree?

Indeed, this is an educated estimate, as no direct data is available (no one ever counted the number of tracheids along a 50 m tall tree).

Line 105: There is no evidence, as far as I know, that cavitation is a correct term and the actual process that leads to embolism. Homogeneous nucleation does not occur in xylem sap and is not relevant. The expansion of a pre-existing bubble is unlikely to be "cavitation", which is the creation of void, with a phase change from liquid to water vapour. Also, what is air-seeding really? This term is used in many

papers, but simply seems to be a term for something that we mechanistically do not understand. The term air-seeding is unclear and not required here.

We used embolism expansion and avoid the cavitation as indeed there is no evidence for spontaneous cavitation

Line 112: xylem tissue (or wood) is a highly complex, anisotropic solid. Therefore, it could be somewhat confusing to apply the term bulk modulus to xylem tissue as this may not contain enough information to cover its full behaviour or resistance to bulk compression.

Yes, this why we performed a sensitivity analysis using a broad range of bulk modulus values (1-10 GPa).

Fig. 2e: The text refers to the xylem pressure after embolism as px + Ka/L, while Kl/L is mentioned in the figure. I also assume that the length of the bubble should be a and not l in Fig. 2e?

px is the initial pressure in adjacent conduit while $K\ell/L$ is the change in pressure resulting from water flowing into the adjacent conduit during the embolus expansion – thus in the figure we only describe the additional pressure. We have changed the notation to ℓ = embolus length and ℓ_{max} = conduit length i.e. maximum length of embolus.

Fig. 3 and general discussion – A very common combination in angiosperms (and some ferns) is the occurrence of vessel and tracheids. The majority of tracheids have pit membranes with a homogeneous thickness. These tracheids are perfectly functional for water transport, and any evolutionary pressure that would result in for evolution of torus-margo pit membranes seems to be lacking. Obviously, the authors do not have to explain why there are tracheids without torus-margo pit membranes, but this combined occurrence of vessels and tracheids is so common that it should be mentioned. Also, please refer to the original reference for claiming that Welwitschia may have vessels and torus-margo pit membranes. These observations of vessels by Carlquist were based on SEM observations of samples treated with strong acids, which is known to convert pit membranes is not artificially large "perforations". So this record should be interpreted more carefully.

We agree that vessels and tracheids commonly co-occur and that tracheids with homogeneous pit membranes can contribute to axial water transport. However, these points are tangential to our specific focus, which is on whether torus-margo pit membranes can operate across vessel-vessel interfaces. The reviewer is also correct regarding *Welwitschia*, we appreciate this correction. We have removed the earlier reference and replace it with *Ephedra*, for which several studies have reported vessels containing torus-margo membranes. We have now also incorporated *Ephedra* into our analysis and discussion, and we cite the relevant supporting studies in the revised manuscript.

Line 152: Vessels in juvenile wood and mature wood are well known to show quantitative differences, and this does not apply to conifers only. The suggestions made here are probably too speculative and could be left out.

We left this section out.

Line 158: Please give more details about how tracheid length was measured. I assume that the Ladell method has been applied?

We directly measured the length of 50 earlywood tracheids from longitudinal samples of each species under a light microscope.

Reviewer #3 (Remarks to the Author):

Overall this is an interesting paper with a novel hypothesis suggesting the failure of angiosperms to utilize the torus-margo pit membrane in the xylem of woody plants. There are a number of anatomical terminology issues throughout. The authors have also missed several key papers that address the same

problem, answer some of the same questions, and either perform the same experiments (Sperry and Tyree 1990) or provide data for which the authors claim does not exist (Zelinka et al. 2015). These are not fatal omissions, and I believe that the inclusion of a discussion of those data (as well as the others cited below) will help to better contextualize the data presented in the manuscript. Most importantly the authors provide a compelling hypothesis, as well as evidence to support it. But, the Zelinka et al. 2015 severely undercuts one of the main claims of novelty and the authors will need to acknowledge it, as well as the data by Sperry and Tyree 1990 that provide estimates on the pressure required to close the torus-margo pit. What is novel here is the proposed mechanism. So, while some of the data presented here are not as novel as the authors have made them out to be, they are generally in line with existing data which can be used to further support their claims.

We agree that there has been a continuous effort to estimate or directly measure the pressure required for torus-margo pit closure, although research in this area remains limited. We now explicitly discuss Sperry & Tyree (1990), Zelinka et al. (2015), along other relevant studies to better contextualize our findings.

Specific comments:

The authors could improve the manuscript considerably by better articulating the conclusions section (the final paragraph before the methods, Lines 148-154). This isn't as strong as it could be. The text at times is not written for a broad audience and a significant knowledge of the plant hydraulics literature is required to fully follow the logic.

There are two problems with the abstract. While I appreciate the purpose of being concise and the constraints of the word limit, it isn't quite as simple as the authors have presented the problem. An important parallel thread in the evolution of hydraulic properties of plants is the ferns, which have tracheids but do not have torus-margo pit membranes. There are a few taxa that reportedly have 'vessels', such as Pteridium (Carlquist makes a note of this), but the fern example (tracheids with homogenous pit membranes) makes for a good comparison to the question addressed by this paper. There's a line (140) that briefly mentions the "dominance of conifers over lycopods and ferns", but there's no reference there and it is a bit misleading in terms of what the authors mean by 'dominance'. In terms of diversity the ferns outnumber the conifers, and lycophytes have been around for a much than conifers. It might be worth qualifying this statement (line 140) in terms of biomass or the evolutionary timescale that the authors are considering.

Second, there are examples of tori in angiosperms, such as in Ulmus and other related taxa (Ref 16). From the anatomical perspective the abstract, as stated, is true but there have been evolutionary attempts at something similar to the torus-margo pit membrane in angiosperms. The authors note this issue (Line 143), but it might be worth considering Coleman CM, Prather BL, Valente MJ, Dute RR & Miller ME. 2004. Torus lignification in hardwoods. IAWA J. 25: 435–447.

Lines 15-17. The authors should provide a reference for the "one-third of water vapor..." statement.

In the rewriting of the introduction this sentence was deleted.

Line 20-21: It would be worth citing Brodribb et al. here since they show a direct connection between hydraulic failure and tissue death: https://doi.org/10.1111/nph.17577

In the rewriting of the introduction this sentence was also removed, it is not directly affecting the results.

Line 23: "that allow water, but not gas, to pass." This is only partially true based on our understanding of air-seeding and the movement of gas bubbles through the xylem. The pit membranes only prevent air movement up until a certain pressure threshold.

We have rewritten the introduction to focus on torus-margo pit membranes and say " Under normal conditions, the margo's large pores allow for relatively unhindered water flow; should an embolism form, the torus seals against the pit aperture, isolating the gas-filled conduit and preventing embolism spread into neighboring conduits".

Lines 25-38: The anatomical terminology in this section is largely incorrect, or at least misleading. The authors have collapsed the pit and the pit membrane into a single unit for some reason, perhaps for simplicity? The first author is well-aware of the correct terminology so this is a curious choice. For example, in line 36 the authors use a mix of terminology to the detriment of their argument "torus-margo pits are 59 times more conductive than membranes". In the first part of this clause they combine the torus-margo pit membrane and the pit itself with this wording, and then only consider the homogenous pit membrane of the angiosperms in the second half of the clause. This is unnecessarily confusing. I think most importantly that the authors go through the manuscript and make careful edits and provide the correct terminology. This is critical because both the pit dimensions (aperture, dimensions of the internal chamber of the pit, etc.) have an influence over the resistance that can be considered separately from the membrane, whereas the authors are sometimes combing the chamber and the membrane, or apparently only considering the membrane in absence of the pit chamber. Paul Schulte's pit chamber and pit membrane modeling are worth considering as well, particularly given that he provides information about the resistance of the margo strand pores(https://doi.org/10.1111/nph.16949;https://doi.org/10.1111/j.1469-8137.2011.03986.x).

We have rewritten the text and corrected the terminology.

Line 41: Again, correct anatomical nomenclature is very important. Here, "suffice to bend the cellulose fibers by which the torus is suspended". The authors are talking about the margo strands. In the preceding sentence they use the term "margo gates", which is not an anatomical term. My recommendation is to use consistent terminology throughout.

We have tried to be consistent with the terminology in the revised manuscript.

Line 41-42: Zelinka et al 2015 measure force-displacement of torus-margo pit membranes (https://doi.org/10.1111/pce.12532). So, this sentence is not entirely accurate.

Correct, we have now included this paper in the manuscript.

Line 42: "we note that vessels are longer". This should be corrected. In general, vessels are longer but there are species with long tracheids (e.g. Sequoia sempervirens) that are as long as some angiosperm vessels. The authors should also provide a reference here showing the relationship between conduit length and volume.

We removed the sentence

Line 43: "If an embolism occurs, the liquid volume occupied by the expanding gas is released into the surrounding tissue, which is under tension." This is stated as fact and unreferenced but I don't think there's any evidence supporting this idea. It is probably true based on current theory, but it is at this point unsubstantiated.

We are not entirely sure what is mean by "unsubstantiated". Since at least Zimmerman (1983), it has been recognized the because water is effectively incompressible, gas expansion during embolism must displace liquid volume from the affected conduit. Subsequent studies (e.g, Lo Gullo & Salleo 1992) have examined the hydraulic effects of water release associated with cavitation events.

To clarify our wording and avoid ambiguity, we have replaced the phrase "surrounding tissue" with a more precise description:

"The fluid forced out of an embolizing conduit must enter neighboring conduits …"

Line 46-47: "the air can migrate across the large gaps in the margo mesh" This statement is in conflict with line 23, see comment above.

The two statements refer to different stages of embolism development. In our revised text, we clarify that when the torus-margo pit is not yet sealed, the high porosity of the margo allows air to pass through, enabling air seeding between conduits. In contrast, once the torus is displaced and seals the pit aperture, the pit effectively isolates the embolized conduit.

Line 48: The authors should provide some rationale behind the <0.1 MPa value. Presumably this is based on Young-Laplace?

Yes, it was – but sentence was removed.

Line 49: The first question is answered by Zelinka et al. 2015, at least for Larix.

Yes, we cite Zelinka et al. 2015 now.

Line 52: "to point where the torus-margo can no longer function." This is vague. Function in what way? Do the authors mean that it is pushed against the pore opening and can no longer conduct water? This hypothesis needs to be better supported.

Yes, in the original manuscript the phrasing was vague. We have rewritten the hypotheses in order to be more explicit.

Lines 55-56: See Zelinka et al. 2015. The authors will need to put their work within the context of this paper, or sufficiently explain why the work presented here is sufficiently different.

We put the work in the context of Zelinka et al. 2015. and use their work to provide support for analysis presented here.

Line 58: Again, Zelinka et al. 2015. Direct measurement of force:displacement of torus-margo pit membranes. Larix and Pinus.
Acknowledged

Line 71: It would be interesting to perform this experiment in species with vestured pits, where the membrane is presumably supported and should not flex as much. See worth by Steven Jansen. E.g. Jansen, Steven, et al. "Vestured pits: do they promote safer water transport?." International Journal of Plant Sciences 164.3 (2003): 405-413. Or, https://doi.org/10.1093/plphys/kiac223 In this second paper (see Fig 5) they model pore constriction size as a function or pressure, which should ultimately influence the conductivity measurements in the present manuscript.

We studied response of the membrane to increasing pressure before. Here we used species with vestured pits and show their membranes did not deflect under increasing pressure: Brendan Choat, Steven Jansen, **Maciej A. Zwieniecki**, Erik Smets, N. Michele Holbrook, Changes in pit membrane porosity due to deflection and stretching: the role of vestured pits, *Journal of Experimental Botany*, Volume 55, Issue 402, July 2004, Pages 1569–1575, https://doi.org/10.1093/jxb/erh173

Line 72: Again, the issue of terminology. "In tori bearing species". It really isn't the torus that is the important part, but the margo strands. The point at which the margo strands flex and allow the torus to seal the pore is the critical mechanism driving the results in Fig. 1. So, calling them 'tori bearing species' is a bit strange.

In the revised manuscript we have removed this terminology.

Line 74-75: "and we attribute it to the gradual occlusion of torus-margo pores." I don't understand why the authors would attribute the reduction in flow to 'occlusion'. The choice of wording here is problematic. Water is flowing through the pores between the margo strands, as well as through the pores on either side of the pit. At the point when the torus covers the pore, with some overlap in terms of the

radius of the torus and the radius of the pore, the whole pit will not allow water to flow through. The water would have to move through the torus at this point, so whether the pores between the margo strands is occluded is not particularly relevant. Also, what would the 'torus-margo' pores be occluded by? Do the authors mean that the pores between the strands shrink?

We reworded it to be to be more precise to "We attribute this nonlinearity to progressive torus displacement and sealing of pit apertures"

Line 76: It would be interesting to compare the result here with that of Zelinka et al. 2015 "The force or calculated pressure needed to displace the pit membrane to an aspirated position was found to be less than 3 μN and is slightly lower than previous calculations of the pressure differences needed to cause aspiration". Furthermore, Zelinka et al. 2015 note in their Discussion section that: "From hydraulic conductivity measurements, researchers have calculated an aspiration pressure difference of between 33 and 470 kPa (Gregory & Petty 1973; Sperry & Tyree 1990; Domec et al. 2006)."

Done

Of particular relevance is the Sperry and Tyree 1990. Their Figure 12 shows essentially the same result as Fig. 1 here. Thus, the data in Fig. 1 are largely confirmatory given that Sperry and Tyree 1990 report changes in conductivity over essentially the same range of applied pressures as the authors do in this paper. They find the same results for two gymnosperms (Abies and Juniperus) and an angiosperm (Acer). Sperry and Tyree 1990's Acer data are more linear than what the authors find here in Fig. 1b, which is interesting as a point of comparison. It is essential that the authors cite this work and put the data into context.

Cited

Line 90 "that the primary function of pits is to limit the spread of gas pockets from one conduit to its neighbors." I would argue that this is primary function of the pit membrane, not the pit. I think this is probably just an issue with terminology.

Fixed

Fig. 3. It would be worth noting in this figure where Welwitschia is situated on this gradient, as well as some of the vessel-less angiosperms, or even the ferns.

We realized *Welwitschia* is not the best example, as it does not have true torus-margo membranes. However, *Ephedra* has been reported to have torus-margo membranes within its vessels, thus we have included *Ephedra* in the revised version.

Line 147. If we look at vessel length distributions, see work by Tyree's group, or Zimmermann and Jeje 1981, we see that the longest vessels make up a very small proportion of the total vessels in a stem. Most of the vessels are skewed toward the shorter end of the spectrum. So, while you might find some exceptionally long vessels in ring-porous species (E.g. Quercus) or linanas with large diameter vessels (E.g. Vitis), those very long vessels (>1m in length) represent a small fraction of the total. The logic proposed here would suggest that there would be a strong selective pressure against torus-margo pit membranes because of the 'longest vessels' would be more vulnerable. This seems like a somewhat weak argument. What may be worth considering is that the ancestral state in the angiosperms is thought to be tracheid based xylem (Hacke et al. 2007; DOI: 10.1086/520724.) In this paper they propose many of the ideas in this present manuscript. "First, vesselless angiosperm wood should have much higher flow resistance than conifer wood because angiosperm tracheids lack low- resistance torus-margo pits. Second, vesselless wood ought to be exceptionally safe from cavitation if the small cumulative area of pits between tracheids confers safety (the pit area hypothesis)."

We have now included both mean and maximum vessel lengths in the analysis. The

Line 156: Taxon names should be in italics.

Done

(Response to reviewers comments (blue italics)
Reviewers' comments (Times New Roman)):

Reviewer #1 (Remarks to the Author):

I reviewed this MS a very long ago. The authors addressed most of my concerns satisfactorily. The manuscript improved in readability, presentation quality and somewhat better clarity in the model. Tiny commen: in the abstract, in line 15, you may wish to remove "-" above the stop.

Thank you for the comment. Little typographical error replaced with dot

Reviewer #2 (Remarks to the Author):

This revised manuscript has improved considerably. The mechanism of how and why aspiration of torus-margo pit membranes is associated with the limited diameter and length of tracheids as compared to multicellular vessels is interesting and supported by the data presented. This finding is novel and attractive, and convincingly presented in Fig. 2-5.

Thank you for the comment

Since pit apertures are hydraulic bottlenecks, there should be a reduced pressure in the pit aperture (but an increase in the flow rate). Bernouilli's principle clearly applies to the pit aperture and pit canal, especially if tracheid walls are fairly thick. This change in pressure may not be relevant, and my understanding is that the model applied assumes that pressure in the pit chamber is similar to the original pressure of the xylem sap in a tracheid before it enters the pit aperture, which would mean that there is no loss of pressure due to energy conservation. It would be useful if the authors could explain this briefly because this is relevant for the generation of the dynamic pressure by embolism propagation.

Good comment. We have clarified this point in the text. We now explicitly state that acceleration of flow through the pit aperture produces a local, reversible reduction in static pressure consistent with Bernoulli's principle, which is represented in the model as a dynamic pressure term ($P_d = \frac{1}{2}\rho {v_{pit}}^2$). We further clarify that this dynamic pressure does not represent an irreversible pressure loss across the pit; rather, static pressure is recovered downstream, while the dynamic pressure term captures the transient force acting on the torus during embolism-driven flow.

Equation 1 and 2: P (pressure) seems to be missing in both equations. Also, how exactly was the pressure calculated here? I assume it is the sum of participating pressures, such as the atmospheric pressure, the hydrostatic pressure, and the capillary pressure (which is dependent on surface tension). This needs to be explained.

Thank you, of course phe pressure (P) should be in the equation – during many rewrites it was lost. We have added it in the revised version.

Further evidence to the hypothesis could be provided by anatomy, and the authors should have

done careful anatomical observations. Firstly, as accurately emphasized in the paper, I fully agree that the magnitude of the dynamic pressure created by embolism propagation depends strongly on the fraction of the conduit wall occupied by bordered pits. The values taken from a single reference should be interpreted carefully as they may not cover the anatomical diversity. They might even be incorrect by up to 1 order of magnitude or more. Based on Pittermann et al. 2006 – the average fraction of conifer tracheid walls that is occupied by bordered pits appears to be 8.6% (or 0.086 ± 0.008, and not 0.00538). The average pit field fraction based on Hacke et al. 2006 was 6.3%, which is similar to the value used by the authors.

Thank you for this comment. We initially used the SOM data from Pittermann paper not the correct values form table in the main text. We only used stem (not root), so in revised version the fraction is 7.35%. We corrected pressure and forces values in the text and redrew in figure.

Secondly, I regret the authors did not take observations of Ephedra. The vessels in species of Ephedra are characterised by forminate perforation plates, which are as large or slightly larger than bordered pits, and clearly different in their development and morphology from scalariform and simple perforation plates. Below is an image showing two neighbouring vessel elements, connected via a perforation plate. Therefore, these conduits are strictly speaking vessels. However, two bordered pits with torus-margo pit membranes can be seen at the bottom; these are bordered pits between a vessel and a tracheid. Whether or not bordered pits in vessels of *Ephedra* show tori or not is unclear.

Point taken. In fact, presented analysis for Ephedra clearly states that pressure required to aspirate torus could be generated only in the tracheid size conduit. We did discuss it in the manuscript. We did not see need for further elaboration on that topic

Also, vesselless angiosperms provide an interesting combination of tracheids with homogeneous pit membranes. However, these taxa likely show extremely long tracheids (compared to tracheids in conifers), with values up to more than 3.5 mm. Obviously, it would be useful to apply the dynamic pressure model of the paper to see if the length would be too high to generate a pit aspiration pressure. Tracheid dimorphism, with "vessel-like" tracheids also occur in these vesselless angiosperms. See for instance: Suzuki M., Joshi L., Fujii T. & Noshiro S. (1991) The anatomy of unusual tracheids in Tetracentron wood. IAWA Bulletin 12, 23–33.

Yes, another interesting point – vessels angiosperm – they most likely could generate pressures required to aspirate torus, but they do not have it. They seemed to combine low conductivity of porous membrane with low conductivity of tracheids – this might also explain their limited distribution to shaded mostly high rainfall areas

Finally, the authors should recognise that many conifers (c.a. 40%) do not possess tracheids with a torus-margo pit membrane (e.g. Jacobsen 2021 Am J Bot), and an interesting question would be to find out if torus aspiration in these taxa may not be needed due to very low vulnerability to embolism, or whether pit membrane aspiration without a pronounced torus would still provide sufficient safety.

Yes – they do not have torus-margo sensu stricta, but almost all have developed margo and central tightly woven cellulose/pectin membrane (with – i.e. structure that has higher hydraulic resitance then margo and can be aspirated – according to Jacobsen type A and B consist of 90% of gumnosperms, and many of type C show also show tightly woven matrix in the center.

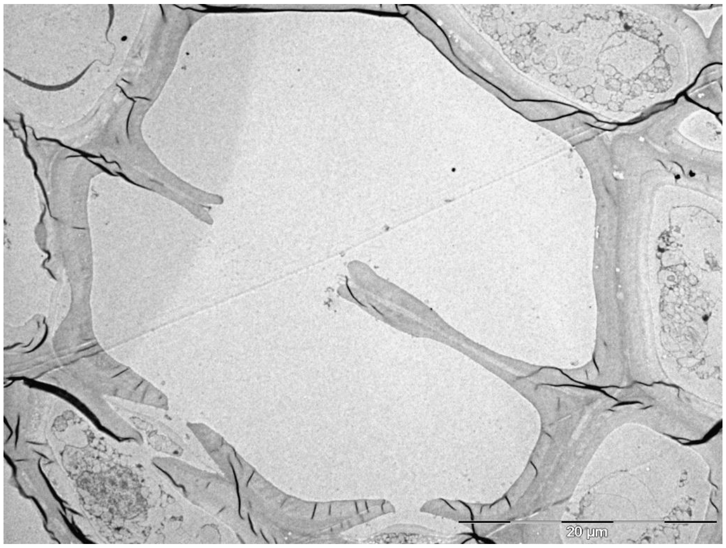

Thank you for addressing most of my previous comments in the reply letter. I agree with the authors that the presence of surfactants would increase the rate of bubble expansion and a modest rise in dynamic pressure to cause torus aspiration. Yet, the polar lipids at gas-liquid interfaces cannot be ignored because they definitively have a strong effect on nanobubble stability and their behaviour in xylem sap (Fig., 4c). As embolism likely develops from pre-existing bubbles, the surface tension of surfactants should at least be mentioned.

Yes – most likely – but the presented data and key message of the paper is not related to stability of bubbles or other methods that plants can use to avoid embolism propagation. The main focus is on discussing surprising lack of torus-margo structures in vessel bearing plants.

Additional comments:
Line 102: what are open pits? Are these bordered pits with non-aspirated pit membranes?

Relaced by 'non-deflected membranes'

Line 109-111: Pit membrane aspiration in angiosperms is much stiffer than conifers and do not easily show any aspiration (Please check Capron et al. 2014 and Carmesin et al. 2023). Also, deformation of the homogeneous pit membrane in angiosperms is well-known, with pit membranes showing an irreversible shrinkage over time (e.g. Sorek et al. 2020; https://doi.org/10.1111/nph.17025). This shrinkage is associated with a reduction of the pit membrane porosity (pore volume fraction) and increased resistance to embolism propagation.

Maybe – but Sorek only show irreversible changes to membrane on seasonal bases not within couple hours of pressure application as observed in our experiments – so we do not see the need

to mention this citations it will distract from the main massage. We described the observation and discus a possible that correspond to timing of the event.

Line 200-202: The terminology used is largely incorrect. There is no cavitation by homogeneous nucleation in xylem. The term air seeding and gas pocket should be avoided here as these are vague, and the concept of air seeding is outdated.

Terminology replaced to reflect mechanism based explanation.

Line 283: what is hydraulic isolation? The presence of ca. 10% of ray parenchyma, and 90% of tracheids makes hydraulic isolation of tracheids in conifers unlikely. Please explain or rewrite.

Reviewer #3 (Remarks to the Author):

The authors have done a good job resolving the issues brought up by the reviewers and I have no major comments at this point. There are typos in the x-axis labels for Figs. 2 and 3 that should be corrected (Trachea should be Tracheid). I think the paper is now much easier to understand it provides an important contribution to our overall understanding of xylem evolution.

Thank you. Typos are corrected

This paper explores how potential pressure reduction associated with embolism formation in conduits can be associated with two major pit membrane types in unicellular tracheids and multicellular vessels. Overall, it is interesting to test if the water released by embolism formation is causing any local pressure increase (i.e. xylem pressure becomes less negative), and how this may affect the valve effect of pit membranes to prevent embolism spreading. The torus-margo aspiration of conifers has been known for a long time, and closure of the pit aperture by a torus is likely an important mechanism for conifers to prevent embolism propagation, while different mechanisms are more likely in angiosperms.

The experimental data suggest that torus-margo aspiration occurs at a pressure difference of 0.25 MPa (or 250 kPa). These values are not novel and largely in line with earlier papers, e.g. Gregory & Petty 1973, Sperry & Tyree 1990, Domec et al. 2006, Beikircher et al. 2010, Bouche et al. 2015 – based on hydraulic measurements, forces required for aspiration were between 8 to 36 µN, and the pressure difference to cause aspiration was between 33 to 470 kPa. A more direct estimation based on force displacement showed that only 3 µN was sufficient to aspirate a torus (Zelinka et al., 2015). Angiosperm pit membranes are clearly much stiffer, and may need a pressure difference above 2 MPa between neighbouring conduits for aspiration (Capron et al. 2014; Carmesin et al. 2023). I miss a more balanced integration of earlier data and literature references, although I fully agree that mechanical properties of pit membranes are not well known.

The expansion of an existing bubble inside a conduit is a more complicated topic than how this is presented here. The bubble size matters, and whether or not the Blake threshold has been reached or not. Bubbles can be stable in a liquid under negative pressure, and the compression pressure on a bubble is determined by the surface tension and the bubble radius. There is strong evidence for the presence of polar lipids in xylem sap, with a dynamic surface tension that can be very low or high. I am surprised to see that surfactants are ignored here as these have an effect on bubble stability, although this makes it hard to model the bubble behaviour and growth. The time of the bubble expansion, and thus the replacement of the liquid from an embolising conduit to a neighbouring conduit should also affect the intercellular pressure drop. Why is time not included in the model? Or is bubble expansion simply assumed to be very fast?

The model is based on an axial, single pathway (Length L), while radial connectivity is not included. Conduits are not only connected axially, but also radially, so neighbouring conduits, but also fibres or parenchyma cells may take up water released by embolised conduits. Why is any radial transport not considered in this model? This may raise questions about the l/L significance (Fig. 2 and 3).

The abstract was rather unclear to me. Without reading the full paper, I did not get the main goal of the paper and the key message. It would be useful to mention that this paper is largely based on a model (Fig. 2), but does not really deal with the "physical conditions necessary for proper embolism isolation", which I found misleading.

Line 23: It is really not correct that xylem conduits (and pit membranes) are airtight because xylem sap is saturated or even oversaturated with gas. Gas molecules can also diffuse through cell wall pores, even if this process is somewhat slow.

The terminology used for xylem anatomy is awkward. The term "pit pore", for instance, is used as a synonym for bordered pit, pit membrane, pit aperture, or a pit membrane pore. I would strongly recommend the authors to use the appropriate terms that are used for the various and geometrically complex openings in secondary cell walls of conduits.

Line 83: Please explain more clearly why -3.4 MPa is taken as xylem pressure for conifers. Is this the average xylem pressure of many conifer species on an average day? This value seems rather negative, and is probably the pressure needed to create embolism. Please explain this more clearly.

Line 86 and 88: Where do these number come from? Any references, or is this a rough estimation about the number of tracheids/vessels in a single axial pathway of an average sized tree?

Line 105: There is no evidence, as far as I know, that cavitation is a correct term and the actual process that leads to embolism. Homogeneous nucleation does not occur in xylem sap and is not relevant. The expansion of a pre-existing bubble is unlikely to be "cavitation", which is the creation of void, with a phase change from liquid to water vapour. Also, what is air-seeding really? This term is used in many papers, but simply seems to be a term for something that we mechanistically do not understand. The term air-seeding is unclear and not required here.

Line 112: xylem tissue (or wood) is a highly complex, anisotropic solid. Therefore, it could be somewhat confusing to apply the term bulk modulus to xylem tissue as this may not contain enough information to cover its full behaviour or resistance to bulk compression.

Fi.g. 2e: The text refers to the xylem pressure after embolism as px + Ka/L, while Kl/L is mentioned in the figure. I also assume that the length of the bubble should be a and not l in Fig. 2e?

Fig. 3 and general discussion – A very common combination in angiosperms (and some ferns) is the occurrence of vessel and tracheids. The majority of tracheids have pit membranes with a homogeneous thickness. These tracheids are perfectly functional for water transport, and any evolutionary pressure that would result in for evolution of torus-margo pit membranes seems to be lacking. Obviously, the authors do not have to explain why there are tracheids without torus-margo pit membranes, but this combined occurrence of vessels and tracheids is so common that it should be mentioned. Also, please refer to the original reference for claiming that Welwitschia may have vessels and torus-margo pit membranes. These observations of vessels by Carlquist were based on SEM observations of samples treated with strong acids, which is known to convert pit membranes inot artificially large "perforations". So this record should be interpreted more carefully.

Line 152: Vessels in juvenile wood and mature wood are well known to show quantitative differences, and this does not apply to conifers only. The suggestions made here are probably too speculative and could be left out.

Line 158: Please give more details about how tracheid length was measured. I assume that the Ladell method has been applied?

This revised manuscript has improved considerably. The mechanism of how and why aspiration of torus-margo pit membranes is associated with the limited diameter and length of tracheids as compared to multicellular vessels is interesting and supported by the data presented. This finding is novel and attractive, and convincingly presented in Fig. 2-5.

Since pit apertures are hydraulic bottlenecks, there should be a reduced pressure in the pit aperture (but an increase in the flow rate). Bernouilli's principle clearly applies to the pit aperture and pit canal, especially if tracheid walls are fairly thick. This change in pressure may not be relevant, and my understanding is that the model applied assumes that pressure in the pit chamber is similar to the original pressure of the xylem sap in a tracheid before it enters the pit aperture, which would mean that there is no loss of pressure due to energy conservation. It would be useful if the authors could explain this briefly because this is relevant for the generation of the dynamic pressure by embolism propagation.

Equation 1 and 2: P (pressure) seems to be missing in both equations. Also, how exactly was the pressure calculated here? I assume it is the sum of participating pressures, such as the atmospheric pressure, the hydrostatic pressure, and the capillary pressure (which is dependent on surface tension). This needs to be explained.

Further evidence to the hypothesis could be provided by anatomy, and the authors should have done careful anatomical observations. Firstly, as accurately emphasized in the paper, I fully agree that the magnitude of the dynamic pressure created by embolism propagation depends strongly on the fraction of the conduit wall occupied by bordered pits. The values taken from a single reference should be interpreted carefully as they may not cover the anatomical diversity. They might even be incorrect by up to 1 order of magnitude or more. Based on Pittermann et al. 2006 – the average fraction of conifer tracheid walls that is occupied by bordered pits appears to be 8.6% (or 0.086 ± 0.008, and not 0.00538). The average pit field fraction based on Hacke et al. 2006 was 6.3%, which is similar to the value used by the authors.

Secondly, I regret the authors did not take observations of Ephedra. The vessels in species of Ephedra are characterised by forminate perforation plates, which are as large or slightly larger than bordered pits, and clearly different in their development and morphology from scalariform and simple perforation plates. Below is an image showing two neighbouring vessel elements, connected via a perforation plate. Therefore, these conduits are strictly speaking vessels. However, two bordered pits with torus-margo pit membranes can be seen at the bottom; these are bordered pits between a vessel and a tracheid. Whether or not bordered pits in vessels of *Ephedra* show tori or not is unclear. Also, vesselless angiosperms provide an interesting combination of tracheids with homogeneous pit membranes. However, these taxa likely show extremely long tracheids (compared to tracheids in conifers), with values up to more than 3.5 mm. Obviously, it would be useful to apply the dynamic pressure model of the paper to see if the length would be too high to generate a pit aspiration pressure. Tracheid dimorphism, with "vessel-like" tracheids also occur in these vesselless angiosperms. See for instance: Suzuki M., Joshi L., Fujii T. & Noshiro S. (1991) The anatomy of unusual tracheids in Tetracentron wood. IAWA Bulletin 12, 23–33.

Finally, the authors should recognise that many conifers (c.a. 40%) do not possess tracheids with a torus-margo pit membrane (e.g. Jacobsen 2021 Am J Bot), and an interesting question would be to find out if torus aspiration in these taxa may not be needed due to very low vulnerability to embolism, or whether pit membrane aspiration without a pronounced torus would still provide sufficient safety.

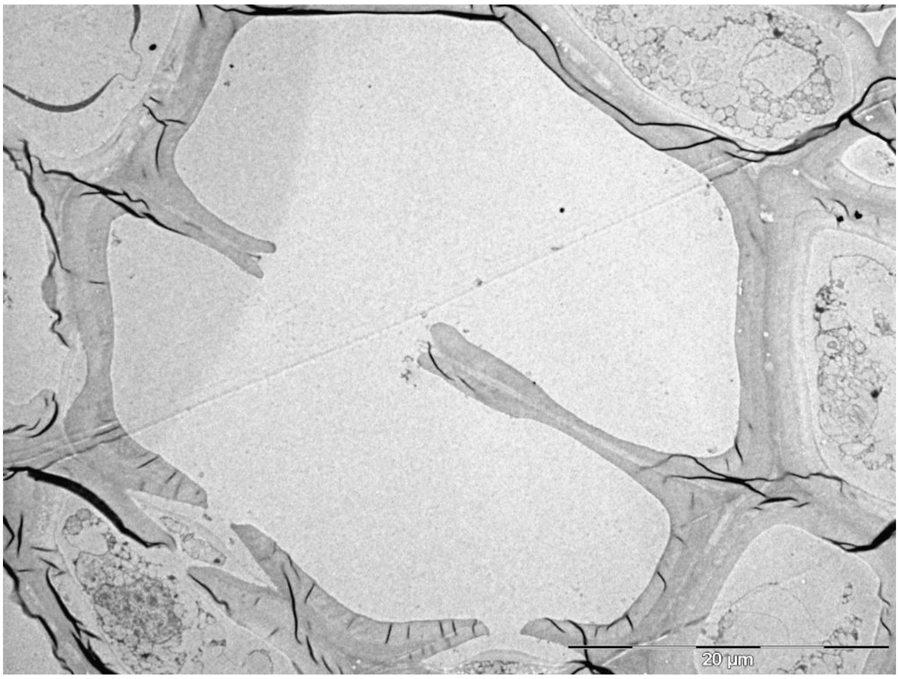

Thank you for addressing most of my previous comments in the reply letter. I agree with the authors that the presence of surfactants would increase the rate of bubble expansion and a modest rise in dynamic pressure to cause torus aspiration. Yet, the polar lipids at gas-liquid interfaces cannot be ignored because they definitively have a strong effect on nanobubble stability and their behaviour in xylem sap (Fig., 4c). As embolism likely develops from pre-existing bubbles, the surface tension of surfactants should at least be mentioned.

Additional comments:

Line 102: what are open pits? Are these bordered pits with non-aspirated pit membranes?

Line 109-111: Pit membrane aspiration in angiosperms is much stiffer than conifers and do not easily show any aspiration (Please check Capron et al. 2014 and Carmesin et al. 2023). Also, deformation of the homogeneous pit membrane in angiosperms is well-known, with pit membranes showing an irreversible shrinkage over time (e.g. Sorek et al. 2020; https://doi.org/10.1111/nph.17025). This shrinkage is associated with a reduction of the pit membrane porosity (pore volume fraction) and increased resistance to embolism propagation.

Line 200-202: The terminology used is largely incorrect. There is no cavitation by homogeneous nucleation in xylem. The term air seeding and gas pocket should be avoided here as these are vague, and the concept of air seeding is outdated.

Line 283: what is hydraulic isolation? The presence of ca. 10% of ray parenchyma, and 90% of tracheids makes hydraulic isolation of tracheids in conifers unlikely. Please explain or rewrite.
